# Critical shortfalls in the management of PBC: Results of a UK-wide, population-based evaluation of care delivery

**DOI:** 10.1016/j.jhepr.2023.100931

**Published:** 2023-10-16

**Authors:** Nadir Abbas, Rachel Smith, Steven Flack, Vikram Bains, Richard J. Aspinall, Rebecca L. Jones, Laura Burke, Douglas Thorburn, Michael Heneghan, Andrew Yeoman, Joanna Leithead, Conor Braniff, Andrew Robertson, Chris Mitchell, Collette Thain, Robert Mitchell-Thain, David Jones, Palak J. Trivedi, George F. Mells, Laith Alrubaiy, UK-PBC audit group

**Affiliations:** 1 National Institute for Health and Care Research (NIHR) Birmingham Biomedical Research Centre (BRC), Centre for Liver and Gastrointestinal Research, University of Birmingham, Birmingham, UK; 2 Liver Unit, University Hospitals Birmingham Queen Elizabeth, Birmingham, UK; 3 Institute of Immunology and Immunotherapy, University of Birmingham, Birmingham, UK; 4 Academic Department of Medical Genetics, University of Cambridge, Cambridge, UK; 5 Cambridge Liver Unit, Cambridge University Hospitals NHS Foundation Trust, Cambridge, UK; 6 Department of Gastroenterology and Hepatology, Portsmouth Hospitals University NHS Trust, Queen Alexandra Hospital, Portsmouth, UK; 7 Department of Hepatology, Leeds Teaching Hospitals NHS Trust, Leeds, UK; 8 Department of Hepatology and Liver Transplantation, Royal Free Hospital NHS Foundation Trust, London, UK; 9 Institute of Liver Studies, King’s College Hospital NHS Foundation Trust, Denmark Hill, London, UK; 10 Aneurin Bevan University Health Board, Newport, UK; 11 Department of Hepatology, Forth Valley Royal Hospital, Larbert, UK; 12 Department of Hepatology, Regional Liver Unit, Belfast Health and Social Care Trust, Belfast, UK; 13 Department of Hepatology, Glasgow Royal Infirmary, Glasgow, UK; 14 PBC Foundation, Edinburgh, UK; 15 Global Liver Institute, UK; 16 Liver Unit, Newcastle Upon Tyne Hospitals NHS Foundation Trust, Newcastle, UK; 17 NIHR Newcastle Biomedical Research Centre, Newcastle University, Newcastle, UK; 18 Institute of Applied Health Research, University of Birmingham, Birmingham, UK; 19 Department of Gastroenterology, St Mark’s Hospital and Academic Institute, London, UK; 20 Swansea University Medical School, Swansea, UK

**Keywords:** Adherence, Autoimmune liver disease, Bezafibrate, Fenofibrate, Guideline, Liver transplantation, Obeticholic acid, Second-line therapy, Service evaluation, Surveillance

## Abstract

**Background & Aims:**

Guidelines for the management of primary biliary cholangitis (PBC) were published by the British Society of Gastroenterology in 2018. In this study, we assessed adherence to these guidelines in the UK National Health Service (NHS).

**Methods:**

All NHS acute trusts were invited to contribute data between 1 January 2021 and 31 March 2022, assessing clinical care delivered to patients with PBC in the UK.

**Results:**

We obtained data for 8,968 patients with PBC and identified substantial gaps in care across all guideline domains. Ursodeoxycholic acid (UDCA) was used as first-line treatment in 88% of patients (n = 7,864) but was under-dosed in one-third (n = 1,964). Twenty percent of patients who were UDCA-untreated (202/998) and 50% of patients with inadequate UDCA response (1,074/2,102) received second-line treatment. More than one-third of patients were not assessed for fatigue (43%; n = 3,885) or pruritus (38%; n = 3,415) in the previous 2 years. Fifty percent of all patients with evidence of hepatic decompensation were discussed with a liver transplant centre (222/443). Appropriate use of second-line treatment and referral for liver transplantation was significantly better in specialist PBC treatment centres compared with non-specialist centres (*p* <0.001).

**Conclusions:**

Poor adherence to guidelines exists across all domains of PBC care in the NHS. Although specialist PBC treatment centres had greater adherence to guidelines, no single centre met all quality standards. Nationwide improvement in the delivery of PBC-related healthcare is required.

**Impact and implications:**

This population-based evaluation of primary biliary cholangitis, spanning four nations of the UK, highlights critical shortfalls in care delivery when measured across all guideline domains. These include the use of liver biopsy in diagnosis; referral practice for second-line treatment and/or liver transplant assessment; and the evaluation of symptoms, extrahepatic manifestations, and complications of cirrhosis. The authors therefore propose implementation of a dedicated primary biliary cholangitis care bundle that aims to minimise heterogeneity in clinical practice and maximise adherence to key guideline standards.

## Highlights

• In this population-based evaluation of clinical care delivery, data was accrued from nearly 9,000 patients with PBC.
• Except for using ursodeoxycholic acid as first-line therapy, adherence to guideline standards was poor in every assessed domain.
• Less than 50% and 65% of patients meeting criteria for second-line treatment or liver transplant assessment, respectively, were appropriately referred.
• Assessment of symptoms and extra-hepatic manifestations were performed in less than 60% of all patients.

## Graphical abstract

**Figure:**
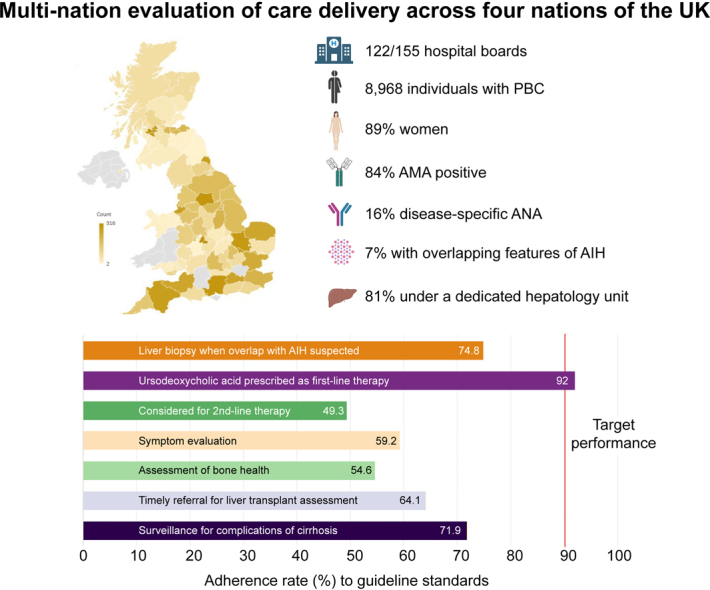

## Introduction

An estimated 25,000 people in the UK live with primary biliary cholangitis (PBC),^1^ a cholestatic liver disease which progresses to cirrhosis and its attendant complications in many patients. Although rare, PBC accounts for approximately one-tenth of liver transplant (LT) activity in the UK.^2^ Progression to end-stage liver disease and the need for transplantation can be mitigated by optimal use of disease-modifying therapies.[3], [4], [5]

In 2018, the British Society of Gastroenterology (BSG) updated its guidelines on the management of PBC. These guidelines describe three pillars of care: (1) ‘Treat & Risk Stratify’, emphasising the importance of optimally dosed first-line therapy, with timely initiation of second-line therapy (SLT) in patients with inadequate ursodeoxycholic acid (UDCA) response; (2) ‘Stage & Survey’, highlighting the value of surveillance for hepatocellular carcinoma (HCC) in patients with cirrhosis, screening for gastroesophageal varices in those with clinically significant portal hypertension, and prompt LT assessment for those with hepatic decompensation; and (3) ‘Actively Manage’, stressing the need to evaluate and treat symptoms such as pruritus, and associated conditions such as osteoporosis. Additionally, the BSG guidelines included several service standards that were intended to be a benchmark for PBC-related healthcare (Table 1).

**Table 1:** Summary of BSG Service Standards.

Service standard	Target
All patients with suspected PBC should have an abdominal ultrasound as part of their baseline assessment.	90%
All patients should be offered first-line treatment with UDCA at 13–15 mg/kg/day.	90%
Individualised risk stratification using biochemical response indices is recommended following 1 year of UDCA therapy.	80%
All patients should be evaluated for the presence of symptoms, in particular fatigue and pruritus.	90%
All patients with a bilirubin >50 μmol/L or evidence of decompensated liver disease should be discussed with a hepatologist linked to a transplant programme (within 3 months).	90%
All patients should have a risk assessment for osteoporosis (within the last 5 years).	80%
When overlap with autoimmune hepatitis is suspected, liver biopsy with expert clinicopathological assessment should be undertaken to support diagnosis.	90%

BSG, British Society of Gastroenterology; PBC, primary biliary cholangitis; UDCA, ursodeoxycholic acid.

We recently piloted an audit of PBC-related healthcare in 11 National Health Service (NHS) hospitals across the UK, which showed that none of the participating centres had achieved the BSG standards.^6^ In the current study, recognising the failings were likely to be systemic, we extended our evaluation of PBC-related healthcare to NHS centres throughout the UK. We aimed to (1) evaluate overall performance against key service standards; (2) compare performance between specialist PBC treatment centres and non-specialist centres; and (3) compare prescribing rates for SLT across the constituent nations of the UK, which have adopted different models for SLT delivery.

## Patients and methods

### Selection of benchmark standards

We convened a working group in August 2020, consisting of hepatologists and gastroenterologists, patient representatives, and a data manager. To ensure adequate representation, we selected physicians from specialist and non-specialist centres across the four nations of the UK (Data S1). Following a consensus voting process, the working group agreed on the scope and standards of the audit, which were adopted from the service standards listed in the 2018 BSG PBC guidelines, the 2016 National Institute for Health and Care Excellence (NICE) guidelines on cirrhosis management, and the 2015 BSG guidelines on varices in cirrhosis^7^ (Table S1). Comparison of these standards with international PBC guidelines published by the European Association for Study of the Liver (EASL) and other international bodies is provided in Table S2.

### Site invitation and case finding strategy

All NHS acute trusts in the UK were invited to participate (^a^n acute trust is an organisational unit that provides secondary care services in the NHS). To maximise study participation, we established a national trainee network, consisting of junior doctors enrolled in specialty training (Data S1). Following registration of the audit, the local audit team (consisting of a consultant hepatologist or gastroenterologist and one or more specialty trainees) used active case-finding to identify patients with PBC under current follow-up. Active case-finding included one or more of the following strategies: (1) interrogation of hospital clinical coding databases (inpatient or outpatient) for individuals with an International Classification of Diseases (ICD)-10 code for PBC (K74.3), including those with concomitant codes for autoimmune hepatitis (AIH) (K73.2 and K75.4); (2) interrogation of immunology laboratory databases for patients with PBC-specific autoantibodies; (3) searching of histopathology laboratory databases for patients with liver biopsies compatible with PBC; and (4) screening of gastroenterology or hepatology departmental case notes and databases for patients with PBC, including those with features of AIH. For patients under follow-up in a local (non-specialist) centre but referred to a regional (specialist) centre for SLT, data were captured from the local centre to avoid duplication. Liver transplant recipients were excluded.

### Data collection and quality control

We created an electronic case report form (eCRF) using the Research Electronic Data Capture platform (REDCap; Vanderbilt University, Nashville, TN, USA), a secure web-based application licensed by the University of Cambridge (Cambridge, UK). Data capture included the patient’s sex, age group, and details about their PBC including diagnosis, first- and second-line treatment, risk of disease progression (defined as an abnormal bilirubin and/or alkaline phosphatase [ALP]>1.67 × upper limit of normal [ULN] after ≥12 months of treatment), symptom assessment, fracture risk assessment, HCC and varices surveillance, and whether a patient met referral criteria for transplant assessment. No patient identifiable details were collected. We provided audit teams with a user guide to support data entry and define data fields (Table S3). Following the submission of eCRFs, the data manager checked for omissions and resolved these with the local audit team. The period of data capture extended from 1 January 2021 until 31 March 2022. The data were subsequently downloaded from REDCap for analysis.

### Data and statistical analysis

For each participating centre, we determined adherence to the audit standards. We then compared adherence according to type of centre (specialist *vs.* non-specialist centre), geographical region and model of SLT delivery, based on the following considerations unique to the UK.

**Fig. 1:**
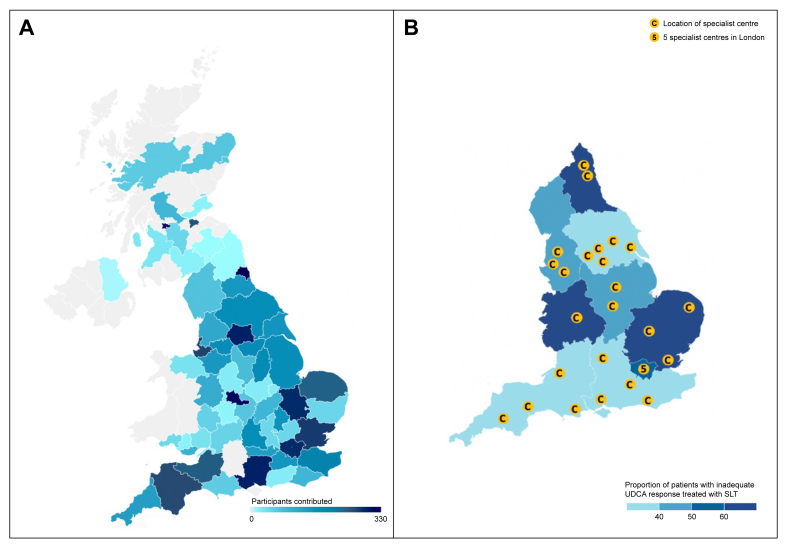
Regions contributing to the PBC audit and location of specialist centres in England. Choropleth map indicating (A) the number of participants contributed by region, and (B) the location of regional specialist centres in England responsible for prescribing second-line treatment. SLT, second-line therapy; UDCA, ursodeoxycholic acid.

The four constituent nations of the UK (England, Scotland, Wales, and Northern Ireland) have adopted distinct models for the delivery of SLT for PBC. In England, patients eligible for SLT are referred to a regional multi-disciplinary team (MDT), located in a specialist hepatobiliary centre (‘specialist centre’) that is networked to neighbouring, non-specialist hospitals (Fig. 1). The specialist centre is responsible for the approval of SLT and the prescription of obeticholic acid (OCA). In Wales and Northern Ireland, SLT is decided by a national MDT. This contrasts from Scotland, where SLT can be prescribed by any Hepatologist or Gastroenterologist, without input from an MDT.

There are seven LT-centres located in the UK: six in England, one in Scotland, and none in Wales or Northern Ireland. Patients eligible for LT in regions that do not contain an LT-centre must be referred to centres in other regions.

Continuous variables are represented by the median value and IQR. We used the non-parametric Mann–Whitney *U* test to look for differences between two discrete groups, and the Kruskal–Wallis test (with Dunn’s *post-hoc* correction) for more than two groups. Categorical variables are represented by numbers and percentages (%). We used a Χ^2^ or Fisher’s exact test to identify differences between groups, and the odds ratios (ORs) with 95% CIs to quantify those differences. We considered a value of *p* <0.05 in a two-sided test to be statistically significant. Analyses were conducted using SPSS Statistics v24.0 (SPSS Inc., Chicago, IL, USA).

### Patient and public involvement

The PBC Foundation (www.pbcfoundation.org.uk) and Global Liver Institute (GLI) (globalliver.org) provided patient and public involvement, nominating two members and one member, respectively, to be on the working group. Patient representatives were involved in all aspects of the project, including the selection of audit standards, design of the eCRF, interpretation of the data, and writing of the manuscript.

### Ethics and governance

The study was a service evaluation. No identifiable patient information was collected. Day-to-day management of individual patients was not affected. The study was registered with the hospital audit office of each participating site before data collection. The NHS code of confidentiality was followed by all sites.^8^

## Results

### Characteristics of the study population

We gathered data on 8,968 patients with PBC who were under follow up in 122 NHS centres across the UK. Most patients were women aged ≥50 years (n = 7,085; 79%). Eighty-one percent of patients (n = 7,263) were followed-up in a hepatology clinic; the remainder by gastroenterology (Table 2).

**Table 2:** Characteristics of audit patients.

Patient characteristics	n (%)
Total patients	8,937
Female	7,941 (88.9)
Male	996 (11.1)
Age group (years)	
20–29	26 (0.3)
30–39	163 (1.8)
40–49	732 (8.2)
50–59	1,969 (22.0)
60–69	2,534 (28.4)
70–79	2,545 (28.5)
>80	968 (10.8)
Autoantibodies	
AMA	7,518 (84.1)
PBC-specific ANA	1,459 (16.3)
PBC/AIH-overlap	679 (7.6)
Clinic	
Hepatology	7,263 (81.3)
General gastroenterology	1,674 (18.7)
Nation	
England	7,690 (86.0)
Northern Ireland	57 (0.6)
Scotland	953 (10.7)
Wales	237 (2.7)

AIH, autoimmune hepatitis; AMA, anti-mitochondrial autoantibody; ANA, anti-nuclear autoantibody; PBC, primary biliary cholangitis.

### Liver biopsy

**Fig. 2:**
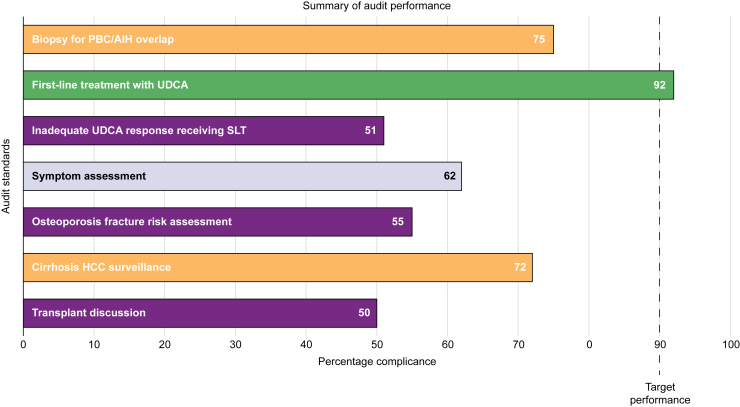
Summary of overall audit performance. This histogram illustrates overall performance for each audit standard under evaluation across the four nations of the UK. AIH, autoimmune hepatitis; HCC, hepatocellular carcinoma; PBC, primary biliary cholangitis; SLT, second-line therapy; UDCA, ursodeoxycholic acid.

Almost one-third of patients underwent a liver biopsy (n = 2,856; 32%); of these, 68% (n = 1,945) had cholestatic biochemistry and either positive anti-mitochondrial autoantibodies (AMAs) and/or PBC-specific anti-nuclear autoantibodies (ANAs). As the use of liver biopsy may have declined since the release of EASL guidelines in 2017 and BSG guidelines in 2018, we compared rates before and after 2017: in all, 35% of patients diagnosed with PBC before 2017 had undergone a liver biopsy (2,239 of 6,446 patients), compared with 25% diagnosed since (617 of 2,491 patients) (*p* <0.001). Conversely, one in four patients reported to have PBC/AIH-overlap syndrome (n = 508) had not undergone histological evaluation (Fig. 2 and Table S4).

### Disease-modifying treatment

Almost 90% of patients were treated with UDCA (n = 7,864), with patient weight and dose of UDCA available for n = 6,053. Nearly one-third of patients (n = 1,850) received a sub-optimal dose (*<*13 mg/kg/day), of whom 48% had an ALP value above the ULN (and 13%, an ALP >1.67 × ULN). In patients who were not treated with UDCA (n = 998), the reason was clearly documented in 72% (n = 721), the most common being drug intolerance (n = 362). Only one in five UDCA-untreated patients were prescribed an alternative second-line agent (n = 202). Amongst patients on UDCA monotherapy for at least 12 months, 2,102 had evidence of inadequate UDCA response; only half were prescribed SLT (n = 1,074; 51%). The choice of SLT therapy was split equally between OCA (n = 572) and fibric acid derivatives (bezafibrate or fenofibrate; n=571), with a small proportion of patients receiving both (n = 83), and a minority being treated with an alternative agent (n = 68). In England, Wales, and Northern Ireland, 73% of patients (682/933) with inadequate UDCA response who had not received SLT, had not been referred to the regional MDT. Sixty percent of these patients were under the age of 70 years.

### Assessment of symptoms and extrahepatic manifestations

More than one-third of patients had not been assessed for fatigue (n = 3,885; 43%) or pruritus (n = 3,415; 38%) in the previous 24 months. Of those reported to have pruritus (n = 1,895), 67% received treatment, most often colestyramine (41%), antihistamines (30%), or rifampicin (17%). Only 55% of patients had undergone fracture risk assessment in the previous 5 years (n = 4,883). One-third of those assessed were deemed to have a clinically significant risk of fracture (n = 1,566; 32%), of whom 92% had received appropriate therapy to reduce this risk. Most patients who had not undergone fracture risk assessment were women above the age of 50 years (n = 2,596; 75%).

### Discussion with a liver transplant centre

At the time of data collection, 443 patients had a serum total bilirubin >50 μmol/L or other features of decompensated cirrhosis; 50% of these patients had not been discussed with a transplant centre. Taking age >70 years to be an arbitrary exclusion criterion for LT, 36% of patients with hepatic decompensation (93/259) had not been referred for transplant assessment despite the advanced nature of their liver disease.

### Surveillance for HCC and gastroesophageal varices

Overall, 1,947 patients were reported to have cirrhosis. Of these, 28% (n = 548) had not undergone ultrasound surveillance for HCC in the previous 6 months. In total, 905 patients were reported to have clinically significant portal hypertension, of whom 23% (n = 210) had not undergone endoscopic variceal surveillance in the previous 3 years. There was no clear documentation to account for the delay in ultrasound and endoscopic surveillance in 64% (n = 348) and 48% (n = 100) of patients, respectively. The COVID-19 pandemic was reported to account for the respective delays in just 8% and 5% of patients.

### Variation in PBC-related healthcare across the UK

We then compared prescribing rates for SLT across the constituent nations. As very few patients had received SLT in Northern Ireland, data from this nation were excluded from the analysis. Despite different models of SLT delivery between nations, there were no differences in the proportion of eligible patients prescribed SLT in England (51%), Scotland (52%) or Wales (50%) (Table S5). There was, however, a difference in choice of therapy. In England and Wales, OCA was prescribed to 55% and 54% of eligible patients, respectively, whereas in Scotland, OCA was prescribed in only 16% eligible patients (*p* <0.001); the remainder received fibric acid derivatives.

**Fig. 3:**
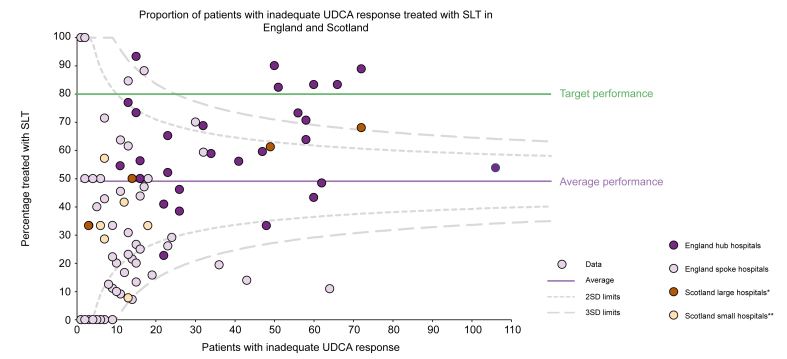
Variation in the prescription of second-line treatment in England and Scotland. Funnel plot displaying the number of patients with inadequate response receiving second-line treatment according to the number of patients with inadequate UDCA response seen by each centre in England and Scotland. ∗Teaching hospital affiliated to a medical school. ∗∗District general hospital. SLT, second-line therapy; UDCA, ursodeoxycholic acid.

In England, eligible patients were more likely to receive SLT if they were followed up in a specialist *vs.* a non-specialised centre (67% *vs.* 30%; OR 4.69, 95% CI 3.82–5.76; *p* <0.001) (Table S6 and Fig. S1). This was also evident in Scotland, where eligible patients were more likely to receive SLT if they were followed up in larger teaching hospitals compared with smaller district general hospitals (63% *vs.* 31%, OR 3.89, 95% CI 2.01–7.78; *p* <0.001) (Fig. 3). Specialist centres were also more likely to ensure UDCA was optimally dosed (74% *vs.* 66%; OR 1.48, 95% CI 1.31–1.68; *p* <0.001); ensure patients with cirrhosis underwent HCC surveillance (72% *vs.* 68%; OR 1.80, 95% CI 1.26–2.58; *p* <0.001); and discuss patients with hepatic decompensation with an LT centre (76% *vs.* 56%, OR 2.49, 95% CI 1.34–4.69, *p* <0.001). They were more likely to assess pruritus (65% *vs.* 58%; OR 1.34, 95% CI 1.22–1.47; *p* <0.001) but no more likely to assess fatigue (57% *vs.* 56%; OR 1.01, 95% CI 0.92–1.11, *p* = 0.82).

**Fig. 4:**
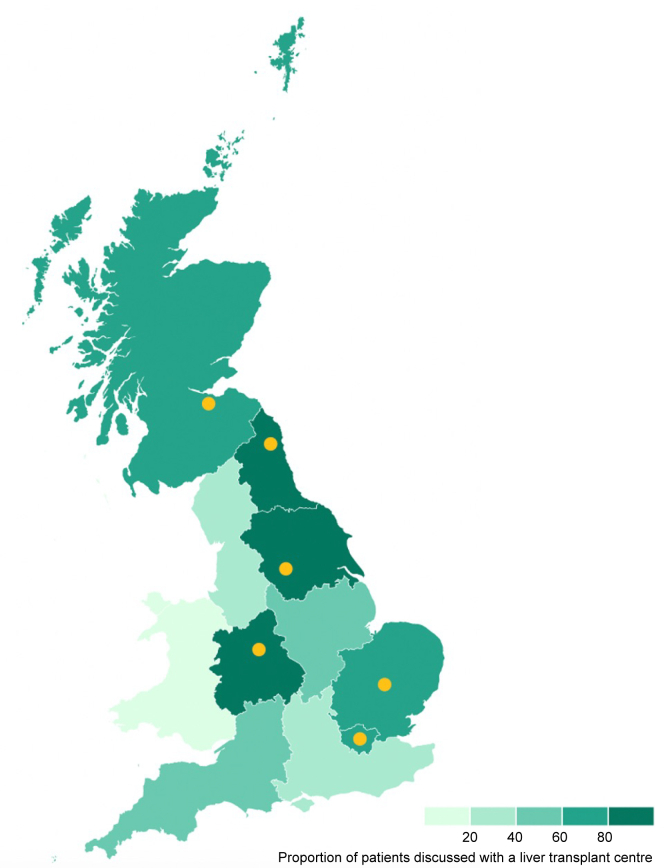
Variation in referral for transplant assessment across three of the four nations in the UK. Choropleth map indicates the proportion of patients in each geographical region referred for liver transplant assessment. Transplant centres highlighted in yellow: from North to South indicate Edinburgh (the only liver transplant unit in Scotland), Newcastle, Leeds, Birmingham, Cambridge and London (note: two liver transplant centres are located in London, the Royal Free Hospital and King’s College Hospital). No liver transplant centres are located in Wales.

We also analysed referral rates for LT in England, Scotland, and Wales. Northern Ireland recorded no cases of hepatic decompensation during the service evaluation period so was not included. Patients eligible for LT were eight times more likely to be referred for transplant assessment if they lived in England or Scotland compared with Wales (52% *vs.* 11%; OR 7.98, 95% CI 1.82–72.6; *p* = 0.001). In England, patients eligible for LT were sevenfold more likely to be referred for LT if they lived in a region containing a LT-centre compared with regions not containing a LT-centre (82% *vs.* 40%; OR 6.99, 95% CI 3.60–13.90; *p* <0.001) (Fig. 4 and Table S7).

## Discussion

PBC is a disease with a clinical and societal burden disproportionate to its prevalence. Although effective medical therapy exists, delays in diagnosis and treatment perpetuate poor outcomes.^9^ Therefore, identifying deficiencies in healthcare has practical implications, and a first step in quality assurance for any clinical service.^10^^,^^11^ In this UK-wide evaluation of PBC healthcare, we identified inadequate adherence to guidelines in all participating centres. Performance was suboptimal in all but one domain. Most striking was the proportion of eligible patients who were not receiving SLT, especially in non-specialist centres. Also striking was the proportion of patients with decompensated cirrhosis who were not referred for LT, particularly in regions without an LT centre.

In all, 50% of patients with inadequate UDCA response had not received SLT. In England, Wales, and Northern Ireland, nearly three-quarters of these patients had not been referred to an MDT, suggesting that the underlying problem is a failure to recognise when SLT is needed. Given that 60% of SLT-eligible but untreated patients were under the age of 70 years, it is unlikely that local centres had considered SLT for patients but deemed them unlikely to benefit owing to life-limiting comorbidity. Moreover, at the time of audit, OCA had only recently been approved by NICE for 3 years, so it is debatable whether non-specialist clinicians would have been best placed to decide against the use of a drug they had never prescribed. In Scotland, half of patients with inadequate UDCA response received SLT despite no requirement for MDT approval, supporting the failure to recognise patients in need of SLT, rather than the MDT, as a barrier to access. Addressing the recognition of patients eligible for SLT is a critical topic for future work.^12^^,^^13^

The probability of referral for LT was lowest amongst geographical regions lacking a transplant centre, suggesting that the national provision of such services is inequitable in terms of access across the UK. This finding mirrors previous observations, wherein serum bilirubin was greater in waitlisted patients with longer travel times to LT centres, consistent with delays in referral.^14^ The issue of how best to enhance the equitable provision of LT services is challenging, but evidently improvements are needed. A regional, multi-disciplinary approach to the management of end-stage liver disease, LT outreach clinics within large gastroenterology units (jointly run by transplant hepatologists and local gastroenterologists), and ease of communication between referring and LT centres all play a role.^15^^,^^16^

Symptoms predict global quality of life for people living with PBC.^17^ To this effect, we found evaluation of symptoms to be inadequate, with lack of recent symptom assessment in over one-third of patients. These findings align with the PBC Foundation patient experience survey, which found that 40% of patients had not been asked about their symptoms during the previous 12 months.^18^ In said survey, nearly half of patients who raised queries about fatigue and a quarter of patients who asked about pruritus, received no advice. The availability of newer, quality-of-life tools provides an opportunity to quantify patient symptoms in routine clinical practice,^19^ and readily identify patients who may benefit from lifestyle modifications, pruritus treatment and clinical trial participation.^20^^,^^21^

The diagnosis of PBC can be made using blood tests alone, supported by clinical history and presenting symptoms.^22^ As such, histological confirmation is not needed except when there is diagnostic doubt.^23^ Overuse of biopsy among patients with classical PBC serology, coupled with underuse in the group being attributed a diagnostic label of PBC/AIH-overlap, can be a result of conflicting statements provided between different guideline documents. For instance, the BSG recommendations on use of liver biopsy state that ‘in PBC a liver biopsy should be done in clinically atypical cases such as failure to respond to UDCA.’^24^ The same guideline document also states that ‘Biopsy “may” also be useful in overlap syndrome’. These statements differ from the BSG PBC guidelines, potentially causing confusion for clinicians. Moreover, strict adherence to the guidelines may lead to some patients undergoing unnecessary and invasive procedures, and consequently delay in initiation of SLT. In turn, lack of histological confirmation in cases of putative PBC/AIH-overlap risks harm, as some patients will receive long-term immunosuppression, despite lack of therapeutic benefit.^25^ In a similar vein, contemporary recommendations for the assessment and management of bone health are lacking in chronic liver disease,^26^ which may have resulted in lack of fracture risk assessment. Ensuring that there is alignment between guidelines that have a broad, more general hepatology remit to those that are PBC-specific may improve adherence to standards and limit variations in clinical practice.

Specialist centres generally had better performance than non-specialist centres, suggesting that familiarity with PBC is important for guideline adherence. It should be noted, however, that specialist centres still demonstrated sub-optimal disease management; no single centre achieved target performance across all domains. Improvement is therefore required across-the-board. Care bundles, which list the essential components of management, have been shown to improve compliance with guidelines.^27^ In the UK, use of the BSG/British Association for the Study of the Liver (BASL) Decompensated Cirrhosis Care Bundle improved standards of care in patients with decompensated cirrhosis within the first 24 h of hospital admission.^28^ A PBC care bundle is a potential solution to improve the delivery of PBC-related healthcare in all centres. In non-specialist centres, familiarity with PBC could be improved through attendance at virtual SLT MDT meetings, providing an opportunity for specialist experience to be shared, and the local cohorting of patients into dedicated clinics. Alongside a care bundle, these changes could facilitate the nationwide improvement required in the management of PBC. The development and implementation of a PBC care bundle forms the second phase of this audit and will be followed by a re-audit of PBC care delivery in selected centres to evaluate the impact of such a bundle on compliance with standards.

A notable limitation of our study is that reasons for non-adherence to guidelines were not captured, and similarly, we did not explore reasons for non-referral to regional specialist PBC MDTs. As such, our study was intended to provide a broad overview of care and identify deficiencies for focused evaluation in future work. The findings of this study have been disseminated to respective centres for reflection, allowing them to identify key areas and adopt strategies at a local level to improve the shortfalls identified. The working group will now focus on implementation of a PBC care bundle followed by a re-evaluation of clinical practice, as part of the wider UK quality improvement drive in liver services.^29^

## Financial support

PT and NA receive institutional support from Birmingham NIHR BRC (National Institute for Health and Care Research Biomedical Research Centre): This paper presents independent research supported by the Birmingham NIHR BRC based at the University Hospitals Birmingham National Health Service Foundation Trust and the University of Birmingham. The views expressed are those of the author(s) and not necessarily those of the National Health Service, the NIHR, or the Department of Health. This study was supported by an unrestricted grant provided by Guts UK.

## Authors’ contributions

Data curation, methodology, statistical analysis, writing the original draft: Lead: NA, RS. Conceptualisation, supporting formal analysis, funding acquisition, supervision and writing original draft: PJT, GFM, LA. Patient and public involvement: CT, RMT. Reviewing and supporting the draft: SF, VB, RJA, RLJ, LB, DT, MH, AY, KJ, CB, AR, CM, DJ.

## Data availability statement

Aggregate data that support the findings of this study are available on request from any of the corresponding authors.

## Conflicts of interest

The authors declare no conflicts of interest.

Please refer to the accompanying ICMJE disclosure forms for further details.
