# Supplementary material for: Critical shortfalls in the management of PBC: Results of a UK-wide, population-based evaluation of care delivery
Source: JHEP Rep. 2023 Oct 16;6(1):100931. doi: 10.1016/j.jhepr.2023.100931 (PMC10711474; doi:10.1016/j.jhepr.2023.100931)
Supplement: Multimedia component 1 [file mmc1.pdf]

Critical shortfalls in the management of PBC: Results of a UK-wide, population-based evaluation of care delivery

Nadir Abbas, Rachel Smith, Steven Flack, Vikram Bains, Richard J. Aspinall, Rebecca L. Jones, Laura Burke, Douglas Thorburn, Michael Heneghan, Andrew Yeoman, Joanna Leithead, Conor Braniff, Andrew Robertson, Chris Mitchell, Collette Thain, Robert Mitchell-Thain, David Jones, Palak J. Trivedi, George F. Mells, Laith Alrubaiy on behalf of the UK-PBC audit group

Table of contents

Supplementary Data S1……………………………………………………………………2

Table S1……………………………………………………………………………………8

Table S2……………………………………………………………………………………9

Table S3…………………………………………………………………………………… 12

Table S4…………………………………………………………………………………… 15

Table S5…………………………………………………………………………………… 17

Table S6…………………………………………………………………………………… 19

Table S7…………………………………………………………………………………… 22

Fig. S1………………………………………………………………………………………26

Supplementary Data S1: Audit Working Group and Trainee Network

Specialist Centres

1. Dr Richard J Aspinall, Queen Alexandra Hospital, Portsmouth, England
2. Dr Conor Braniff, Royal Victoria Hospital, Belfast, Northern Ireland
3. Professor Michael Heneghan, King's College Hospital, London, England
4. Professor David Jones, Freeman Hospital, Newcastle, England
5. Dr Rebecca Jones, St James's University Hospital, Leeds, England
6. Dr George Mells, Addenbrooke's Hospital, Cambridge, England[*]
7. Dr Douglas Thorburn, Royal Free Hospital, London, England
8. Dr Palak Trivedi, Queen Elizabeth Hospital, Birmingham, England*
9. Dr Andrew Yeoman, Royal Gwent Hospital, Newport, Wales

Non-Specialist Centres

10. Dr Laith Alrubaiy, St Mark's Hospital, London, England[*]
11. Dr Joanna Leithead, Forth Valley Hospital, Larbert, Scotland

Patient Representatives

12. Robert Mitchell-Thain, Global Liver Institute
13. Chris Mitchell, PBC Foundation, Edinburgh, Scotland
14. Collette Thain, PBC Foundation, Edinburgh, Scotland

Data Manager

15. Steve Flack, University of Cambridge, Cambridge, England

[*] Project co-lead

Supplementary Data S1 Continued: Trainee Network

Nadir Abbas[1], Basma Ahmed Abdelsalam[2], Samad Abdul[3], Duaa Abduljabbar[4], Anwar Abusrewil[5], Lauren Adams[6], Mohamed Adam[7], Salman Ahmed[8], Jawad Ali[9], Robin Aldridge[10], Tanzil Al Imran[10], Lance Alleyne[11], Laith Alrubaiy[12], Sarah Al-Shakhshir[13], Ismaeel Al-Talib[14], Muhammad Nauman Amin[15], Tanmay Anand[16], Bradley Arms-Williams[17], Fahad Ashraf[18], Muteeb Ashraf[2], Usama Aslam[19], Huma Asmat[20], Zaw Myo Aung[21], Desson Au-Yeung[22], Rosewan Aydogan[23], Nauman Babar[24], Faisal Baig[25,26] , Vikram Bains[27], Opeyemi Bamidele[28], Arindam Banerjee[29], M Fahd Baqai[30], Ezzeldin Barakat[31], Alina Barcan[32], Kris Bennett[30], Mandeep Kaur Bhandal[33], Azad Bhushan[34], Karl Bonello[35], Daniel Bordom[36], Joshua Bower[37], Gary Bray[38], Paul Brennan[39], Rebecca Brown[2], Andrei Brunchi[34], Laura Burke[40], Karen Burns[41], Iona Campbell[24], Roisin Campbell[42], Andra Caracostea[43], M Carbonell[44], Zillah Cargill[45], Jennifer Cathcart[46], Manpreet Chahal[47], Gayatri Chakrabarty[48], Michelle Cheung[49], Anmol Chikhlia[16], Xin Yi Choon[50], Yooyun Chung[51], Jennifer Clancy[49], Emma Coleman[52], Christopher Cooper[53], Zoe Cox[54], Jo Coyle[37], Dominic Crocombe[49], Joanna Crombie[28], Lourdes Cumlat[54], Courtney Davies[16], Janet Dearden[29], Mollie Delaney[1], Bruce Dickson[55], Grace Dolman[29], Hannah Donnelly[56], Rebecca Dunn[2], Louise Eales[57], Jonathan Easaw[58], Mostafa El Gindy[18], Kathy Ellwood[59], Maged Elsaie[11], Ahmed El-Sayed[60], Louise Evans[61], Samantha Farrow[35], Helen Fellows[62], Matthew Foxton[8,44], Keith George[91], Andrew Gerges[63], Ben Giles[92], David Gomez[64], Stephanie Goodman[54], Akshaye Gopaul[16], Sarah Gormley[28], Mahmoud Gouda[43], Aarav Gupta-Kaistha[31], Sarah Guthrie[65], Richard Hackett[8], Andreas Hadjinicolaou[66], Neil Halliday[49], Basem Hamzah[2], Bisam-ul Haq[1], James Hassall[67], Omar Herman[68,69], Mathew Hewitt[17], Amy Hicks[70], Lorraine Hill[71], Htar Htar Hlaing[72], Anna Howard[40], Catherine Hsu[73], Muhammad Javaid Iqbal[63], Jumi Isibor[93], Asem Ismail[94], Jamal W Jamal[48], Joel James[74], Pernia Javid[2], Nasir Javed[31], Dhaarica Jeyanesan[43], Rhiannon Jones[17], Laura Jopson[75], M. Junaid Aleem[76], Yiannis Kallis[29], Sophia Kanwar[58], Syed Karim[38], Christopher Kelly[104], Muhammad Usman Khalid[63], Asif Khan[105], Aria Khani[106], Yun Kim[107], Jade King[49], Meera Kirby[50], Sara Koo[108], Shemin Kovammal[23], Victoria Kronsten[43], Ayo Lagundoye[109], Jonathan Lam[49], Wai Liam Lam[62], Brooke Layard[84], Angela Liaros[110], Thinzar Linn[111], Megan Lofthouse[112], Ruairi Lynch[24], Mohan Madayala[77], Aarani Mahalingam[36], Moawad Mahgoub[78], Abdul Manan[64], Vanessa Martin[79], Ben Masterman[80], Charmaine Matthews[81], Iana Martini[16,82], Maria-Emanuela Maxan[83], Nicola McCallion[84], Matthew McCormack[76], Sarah McDermott131, Hannah McDowell[85], Kate McHugh[86], Stephanie Michail[81], Neil Mistry[87], Dhanoop Mohandas[16], Sean Mole[30], Claudia Moore-Gillon[73], James Morgan[67], James Morgan[88], Sean Morris[1], Jennifer Murray[50], Sruthi Murthy[89], Ruhullah Nasiri[63], Muhammad Ahsan Nasim[31], Rooshi Nathwani[49], Edward Nicholson[89], Gaurav Nigam[90], Murtaza Nomani[1], Kirsty Nixon[116], Laura Ogle[5], Olaku Okeke[97], Olaolu Olabintan[113], Elaine Ong Ming San[114], Josh Orpen-Palmer[115], Michael Orrell[22], Glenda Osuoha[40], Christina Owen[28], Aashish Pandya[49], Ioannis Papamargaritis[117], Maurizio Parker[118], Suvey Perera[119], Alex Perry[120], Robert Perry[73], Rory Peters[22], Naw April Phaw[111], Abimbola Phillips[121], Anju Phoolchund[107], Lushen Pillay[82], Emily Platten[111], Keith Pohl[122], Lucia Possamai[73], Krithivasan Praman[66], Yash Prasad[123], Andrew Punton[124], Abdul Hameed Rahimi[95], Satyasheel Ramful[111], Naomi Ratcliff[49], Zaira Rehman[64], Christy Riggott[125], Andrew Robertson[115], Emma Robinson[39], Simon Rushbrook[79], Nafisa Salih[126], David Samuel[96], Emma Saunsbury[127], Jenny Scott[24], Jayshri Shah[23], Jessica Shearer[128], Abhishek Sheth[89], Harsimranjit Sidhu[73], Abigail Silcock[97,98], Sophie Sinclair[21], Prabhsimran Singh[99], Rohit Sinha[100], Belinda Smith[73], R Sood[94], Chrysoula Spyridou[30],Jennifer Steen[129], Kate Stephen[43], Jason Stevenson[101], Elena Sumanariu[23,130], Nikki Summers[102], Liz Sweeney[81,103], Huey Tan[30], Olivia Taylor[82], Jennifer Tham[115], Amy Thatcher[131], Helen Thompson Jones[61], Qamar Tufail[15], Varun Udayachandran[23], Vithushan Vakeeswarasarma[135], Jessiya Veliyankodan Parambil[90], Mathew

Vithayathil[73], Edvard Volcek[82], Anand Vyas[28], Katy Waddell[42], Gwilym Webb[95], Emily Westlake[49], Aaron Wetten[5], Helen White[93], James Willsmore[132,133], Jenna Wooding[134], John Wye[21], Irvin Yeoh[111], Andrew Yeoman[87], Faisal Zahedi[136], Shakir Zaman[84], Andreea Zotescu[23], Seema Zulfikar[23]

1. Queen Elizabeth Hospital, University Hospitals Birmingham NHS Foundation Trust
2. Royal Surrey County Hospital, Royal Surrey County Hospital NHS Foundation Trust
3. East Surrey Hospital, Surrey and Sussex Healthcare NHS Trust
4. Worcester Royal Hospital, Worcestershire Acute Hospitals NHS Trust
5. Freeman Hospital , Newcastle upon Tyne Hospitals NHS Foundation Trust
6. Leighton Hospital, Mid Cheshire Hospitals NHS Foundation Trust
7. Wrexham Maelor Hospital, Betsi Cadwaladr University Health Board
8. Chelsea & Westminster Hospital, Chelsea and Westminster Hospital NHS Foundation Trust
9. Watford General Hospital, West Hertfordshire Hospitals NHS Trust
10. Torbay Hospital, South Devon Healthcare NHS Foundation Trust
11. Leicester Royal Infirmary, University Hospitals of Leicester NHS Trust
12. Northwick Park Hospital, London North West Healthcare NHS Trust
13. Royal Shrewsbury Hospital, Shrewsbury and Telford Hospital NHS Trust
14. Walsall Manor Hospital, Walsall Healthcare NHS Trust
15. Kettering General Hospital, Kettering General Hospital NHS Foundation Trust
16. Lister Hospital, East and North Hertfordshire NHS Trust
17. University Hospital of Wales, Cardiff and Vale University Health Board
18. King's Mill Hospital, Sherwood Forest Hospitals NHS Foundation Trust
19. Ipswich Hospital, East Suffolk and North Essex NHS Foundation Trust
20. Bedford Hospital, Bedford Hospitals NHS Trust
21. Luton and Dunstable Hospital, Bedfordshire Hospitals NHS Foundation Trust
22. John Radcliffe Hospital, Oxford University Hospitals NHS Trust
23. William Harvey Hospital, East Kent Hospitals University NHS Foundation Trust
24. Royal Infirmary of Edinburgh, NHS Lothian
25. Lincoln County Hospital, United Lincolnshire Hospitals NHS Trust
26. Pilgrim Hospital, United Lincolnshire Hospitals NHS Trust
27. Addenbrooke's Hospital, Cambridge University Hospitals NHS Foundation Trust
28. Musgrove Park Hospital , Taunton and Somerset NHS Foundation Trust
29. Royal London Hospital, Barts Health NHS Trust
30. Derriford Hospital, Plymouth Hospitals NHS Trust
31. Royal Derby Hospital , University Hospitals of Derby and Burton NHS Foundation Trust
32. Royal Hampshire County Hospital, Hampshire Hospitals NHS Foundation Trust
33. Frimley Park Hospital, Frimley Health NHS Foundation Trust
34. North Devon District Hospital, Northern Devon Healthcare NHS Trust
35. Victoria Hospital, Kirkcaldy, NHS Fife
36. Broomfield Hospital, Mid Essex Hospital Services NHS Trust
37. George Eliot Hospital, George Eliot Hospital NHS Trust
38. Southend University Hospital, Southend University Hospital NHS Foundation Trust
39. Ninewells Hospital, NHS Tayside
40. St James's University Hospital, Leeds Teaching Hospitals NHS Trust
41. Royal Lancaster infirmary, University Hospitals of Morecambe Bay NHS Foundation Trust

42. University Hospital Hairmyres, NHS Lanarkshire
43. King's College Hospital, King's College Hospital NHS Foundation Trust
44. West Middlesex University Hospital, Chelsea and Westminster Hospital NHS Foundation Trust
45. Maidstone Hospital, Maidstone and Tunbridge Wells NHS Trust
46. Aberdeen Royal Infirmary, NHS Grampian
47. Royal Stoke University Hospital, University Hospitals of North Midlands NHS Trust
48. East Surrey Hospital, Surrey and Sussex Healthcare NHS Trust
49. Royal Free Hospital , Royal Free London NHS Foundation Trust
50. Queen's Hospital, Barking, Havering and Redbridge University Hospitals NHS Trust
51. St George's University Hospital, St George's University Hospitals NHS Foundation Trust
52. Princess Royal Hospital, Brighton and Sussex University Hospitals NHS Trust
53. University College London Hospital , University College London Hospitals NHS Foundation Trust
54. James Cook University Hospital, South Tees Hospitals NHS Foundation Trust
55. Raigmore Hospital, NHS Highland
56. Gloucestershire Royal Hospital , Gloucestershire Hospitals NHS Foundation Trust
57. Medway Maritime Hospital, Medway NHS Foundation Trust
58. Royal Blackburn Hospital, East Lancashire Hospitals NHS Trust
59. Scunthorpe General Hospital, Northern Lincolnshire and Goole NHS Foundation Trust
60. Northwick Park Hospital, London North West Healthcare NHS Trust
61. Singleton Hospital, Swansea Bay University Health Board
62. Hereford County Hospital, Wye Valley NHS Trust
63. Diana Princess of Wales Hospital, Northern Lincolnshire and Goole NHS Foundation Trust
64. New Cross Hospital, Royal Wolverhampton Hospitals NHS Trust
65. Royal Bolton Hospital, Bolton NHS Foundation Trust
66. Hinchingbrooke Hospital , North West Anglia NHS Foundation Trust
67. Northern General, Sheffield Teaching Hospitals NHS Foundation Trust
68. Stoke Mandeville Hospital, Buckinghamshire Healthcare NHS Trust
69. Wycombe Hospital, Buckinghamshire Healthcare NHS Trust
70. Hull Royal Infirmary, Hull University Teaching Hospitals
71. Royal Alexandra Hospital, NHS Greater Glasgow and Clyde
72. West Suffolk Hospital , West Suffolk NHS Foundation Trust
73. St Mary's Hospital, Imperial College Healthcare NHS Trust
74. Royal Cornwall Hospital, Royal Cornwall Hospitals NHS Trust
75. Hexham General Hospital, Northumbria Healthcare NHS Foundation Trust
76. Warwick General Hospital, South Warwickshire NHS Foundation Trust
77. Royal Preston Hospital, Lancashire Teaching Hospitals NHS Foundation Trust
78. Dumfries and Galloway Royal Infirmary, NHS Dumfries & Galloway
79. Norfolk and Norwich University Hospital, Norfolk and Norwich University Hospitals NHS Foundation Trust
80. Yeovil District Hospital , Yeovil District Hospital NHS Foundation Trust
81. Royal Liverpool Hospital, Liverpool University Hospitals NHS Foundation Trust
82. Princess Alexandra Hospital, Princess Alexandra Hospital NHS Trust
83. Croydon University Hospital, Croydon Health Services NHS Trust
84. Royal Victoria Hospital, Belfast Health and Social Care Trust

85. University Hospital Coventry, University Hospitals Coventry and Warwickshire NHS Trust
86. Queen Elizabeth Hospital, Gateshead Health NHS Foundation Trust
87. Royal Gwent Hospital, Aneurin Bevan University Health Board
88. Sandwell General Hospital , Sandwell and West Birmingham Hospitals NHS Trust
89. Queen's Medical Centre, Nottingham University Hospitals NHS Trust
90. Milton Keynes University Hospital, Milton Keynes Hospital NHS Foundation Trust
91. Torbay Hospital, South Devon Healthcare NHS Foundation Trust
92. Queen Alexandra Hospital, Portsmouth Hospitals NHS Trust
93. Salford Royal Hospital, Salford Royal NHS Foundation Trust
94. Russells Hall Hospital, Dudley Group of Hospitals NHS Trust
95. Peterborough City Hospital, North West Anglia NHS Foundation Trust
96. Royal Glamorgan Hospital, Cwm Taf Morgannwg University Health Board
97. University Hospital of North Durham, County Durham and Darlington NHS Foundation Trust
98. Bishop Auckland Hospital , County Durham and Darlington NHS Foundation Trust
99. Selby War Memorial Hospital, York Teaching Hospital NHS Foundation Trust
100. Sunderland Royal Hospital, South Tyneside and Sunderland NHS Foundation Trust
101. Leighton Hospital, Mid Cheshire Hospitals NHS Foundation Trust
102. University Hospital Aintree, Liverpool University Hospitals NHS Foundation Trust
103. Southport Hospital, Southport & Ormskirk Hospital NHS Trust
104. Forth Valley Royal Hospital, NHS Forth Valley
105. Huddersfield Royal Infirmary, Calderdale And Huddersfield NHS Foundation Trust
106. North Middlesex Hospital, North Middlesex University Hospital NHS Trust
107. University Hospital Southampton, University Hospital Southampton NHS Foundation Trust
108. Cumberland Infirmary, North Cumbria University Hospitals NHS Foundation Trust
109. Northampton General Hospital, Northampton General Hospital NHS Trust
110. Whiston Hospital , St Helens and Knowsley Teaching Hospitals NHS Trust
111. University Hospital of North Tees, North Tees and Hartlepool NHS Foundation Trust
112. Medway Maritime Hospital, Medway NHS Foundation Trust
113. Royal County Sussex Hospital, Brighton and Sussex University Hospitals NHS Trust
114. Pinderfields Hospital, Mid Yorkshire Hospitals NHS Trust
115. Gartnavel General Hospital, NHS Greater Glasgow and Clyde
116. Manchester Royal Infirmary, Manchester University NHS Foundation Trust
117. Arrowe Park Hospital, Wirral University Teaching Hospital NHS Foundation Trust
118. Kingston Hospital, Kingston Hospital NHS Foundation Trust
119. University Hospital Hairmyres, NHS Lanarkshire
120. Bristol Royal Infirmary, University Hospitals Bristol NHS Foundation Trust
121. James Paget Hospital , James Paget University Hospitals NHS Foundation Trust
122. Royal Devon and Exeter Hospital, Royal Devon and Exeter NHS Foundation Trust
123. Countess of Chester Hospital, Countess of Chester Hospital NHS Foundation Trust
124. Borders General Hospital, NHS Borders
125. Harrogate Hospital , Harrogate and District NHS Foundation Trust
126. Royal Berkshire Hospital, Royal Berkshire NHS Foundation Trust
127. Royal United Hospital Bath , Royal United Hospitals Bath NHS Foundation Trust
128. York Hospital, York Teaching Hospital NHS Foundation Trust
129. University Hospital Crosshouse, NHS Ayrshire & Arran
130. Kent and Canterbury Hospital , East Kent Hospitals University NHS Foundation Trust

131. Mount Vernon Hospital, Hillingdon Hospitals NHS Foundation Trust
132. Ealing Hospital, London North West Healthcare NHS Trust
133. Central Middlesex Hospital, London North West Healthcare NHS Trust
134. Royal Bournemouth Hospital, Royal Bournemouth and Christchurch Hospitals NHS Foundation Trust
135. Hemel Hempstead Hospital, West Hertfordshire Hospitals NHS Trust
136. Basildon University Hospital , Basildon and Thurrock University Hospitals NHS Foundation Trust

Table S1: UK-PBC Audit Standards

Audit Standards		Target Performance
1	All patients with suspected PBC should have an abdominal ultrasound as part of their baseline assessment, to exclude alternate aetiologies for cholestasis.	90%
2	All patients with suspected overlap features of autoimmune hepatitis (AIH) should have a liver biopsy with expert clinicopathological assessment to support diagnosis.	90%
3	All patients should receive UDCA as first-line treatment, at a dose of at least 13mg/kg/day.	90%
4	All patients with inadequate UDCA response or UDCA-intolerance should be considered for second-line treatment.	n/s
5	All patients should be evaluated for the presence of symptoms, in particular fatigue and pruritus, to ensure appropriate investigation and treatment.	80% (within the last 24 months)
6	All patients should have risk assessment for osteoporosis to optimise prevention of osteoporotic bone fractures. Treatment and follow-up should be according to national guidelines.	80% (within the last 5 years)
7	All patients with a bilirubin >50 μmol/L or evidence of decompensated liver disease should be discussed with a hepatologist in a transplant centre for timely consideration of liver transplantation.	90% (within 3 months)
8	All patients with cirrhosis should have surveillance for hepatocellular carcinoma (HCC).	n/s
9	All patients with clinically significant portal hypertension should have endoscopy screening for gastro-oesophageal varices.	n/s

Table S2: Comparison of National and International PBC guidelines by Major Societies

Audit Standard	BSG Guidelines 2018[1]	EASL Guidelines 2017[2]	AASLD Guidelines 2018[3]	APASL Guidelines 2021[4]
**Patients suspected to have PBC/AIH overlap syndrome should undergo liver biopsy.**	Overlap with AIH should be recognised as rare and, when suspected, liver biopsy with expert clinicopathological assessment is recommended to make the diagnosis.	PBC with features of AIH should be recognised as rare, and when suspected, liver biopsy with expert clinicopathological assessment, is recommended to make the diagnosis.	Liver biopsy to rule out concomitant AIH or other liver disease should be considered in PBC patients when the alanine aminotransferase activity is more than 5 times the upper limit of normal.	The diagnosis of PBC with AIH features could be made in PBC patients if two of the three following criteria are met: (1) moderate/severe interface hepatitis in liver histology (mandatory); (2) serum ALT/AST more than 5 times ULN; and (3) IgG level more than 1.3 times ULN or presence of ASMA.
**All patients should receive first-line therapy with UDCA at an adequate dose, or documented to be intolerant.**	Patients should be offered therapy with UDCA. UDCA at 13–15mg/kg/day is recommended for first-line use in all patients with PBC.	UDCA at 13–15 mg/kg/day is recommended for first-line use in all patients with PBC.	UDCA in a dose of 13 to 15 mg/kg/day orally is recommended for patients with PBC who have abnormal liver enzyme values regardless of histologic stage.	Oral UDCA (13 – 15mg/kg/day) should be standard therapy for all PBC patients.
**UDCA non-responders should be considered for second-line**	UDCA treated patients with an ALP >1.67x ULN and/or elevated bilirubin < 2 x ULN represent a group of high-risk patients	For patients with an inadequate response to UDCA, or for those intolerant to UDCA, consider the use of OCA.	Patients who are inadequate responders to UDCA should be considered for treatment with OCA, starting at 5 mg/day.	OCA should be added to UDCA therapy for PBC patients with an inadequate response to UDCA, or used in

**therapy with OCA or a fibrate.**	in whom there is randomised controlled trial evidence for the addition of second-line therapy.			monotherapy in those intolerant to UDCA.
**All patients should be assessed for pruritus and fatigue.**	Patients should be evaluated for the presence of symptoms, particularly fatigue and itch.	Patients should be evaluated for the presence of symptoms, particularly pruritus, sicca complex and fatigue.	The symptoms of PBC significantly impair quality of life and do not typically improve with UDCA or OCA treatment. Therefore, they warrant separate evaluation and treatment.	n/a
**All patients should have a risk assessment for osteoporotic fracture.**	Patients with PBC should have a risk assessment for osteoporosis. Treatment and follow-up should be according to national guidelines.	Patients should have a risk assessment for osteoporosis. Treatment and follow-up should be according to national guidelines.	Baseline and regular screening every 2 years using bone mineral density testing is appropriate.	Patients should be evaluated for osteoporosis, especially in postmenopausal women.
**All patients with a bilirubin > 50 should be discussed with a transplant centre.**	Patients with a bilirubin >50 µmol/L or evidence of decompensated liver disease should be discussed with a hepatologist linked to a transplant programme.	Patients with a bilirubin >50 µmol/L (3 mg/dl) or evidence of decompensated liver disease (variceal bleed, ascites, encephalopathy) should be discussed with a hepatologist linked to a transplant programme.	Patients with manifestations of end-stage PBC should be referred for liver transplantation when their Model for End-Stage Liver Disease score exceeds 14.	Liver transplant should be considered in patients with decompensated cirrhosis.

**Patients with cirrhosis should be under surveillance for HCC.**	In patients where cirrhosis is suspected, HCC surveillance should be carried out according to NICE guidelines.	Patients with suspected cirrhosis should have HCC surveillance according to EASL guidelines.	Regular screening for hepatocellular carcinoma with cross-sectional imaging at 6-month intervals is currently advised for patients with cirrhosis.	Close monitoring of HCC is recommended for patients with advanced-stage disease and non-responders to UDCA.
**Patients with clinically significant portal hypertension should be screen for gastro-oesophageal varices.**	Patients with suspected portal hypertension should be screened for gastro-oesophageal varices according to BSG guidelines.	Baveno-VI guidelines for screening and management of varices apply equally to patients with PBC.	Patients with suspected cirrhosis should undergo endoscopic screening for varices at the time of diagnosis.	Patients with features of portal hypertension should be screened for gastroesophageal varices.

1. British Society of Gastroenterology
2. European Association of Study of the Liver
3. American Association for the Study of Liver Diseases
4. Asian Pacific Association for the Study of the Liver

Table S3: **Specific Questions with Indicative Timelines For Data Capture**

What is the patient gender? (male/female)
What is the patient's current age?
Is the patient's weight recorded? (Y/N)
What is the patient weight in Kg?
In which clinic is the patient seen?<br>-Hepatology<br>-General Gastroenterology<br>-General Medicine<br>-Others (please specify)
When was the patient first diagnosed with PBC? (Y/N)
Did/does the patient have persistent elevation of serum ALP? (Y/N)
Does the patient have AMA detectable in serum? (Y/N)
Does the patient have PBC-specific ANA detectable in serum? (Y/N)
Has the patient had an USS of the liver at any point since the time of diagnosis? (Y/N)
Did the patient have an USS of the liver at diagnosis? (Y/N)
Has the patient ever had a liver biopsy? (Y/N)
Was the biopsy compatible with PBC? (Y/N)
Does the patient have PBC/AIH overlap syndrome? (Y/N)
Was the PBC/AIH overlap confirmed by a liver biopsy? (Y/N)
Is the patient currently treated with UDCA?<br>Is the current dose of UDCA recorded? (Y/N)<br>What is the total daily dose (mg/day)<br>Why is the patient not treated with UDCA? (If answered no to)<br>-Not offered by clinicians<br>-Declined by patient<br>-Intolerance of UDCA<br>-Unknown<br>-Other (Please specify?)
Was the patient referred to SLT MDT for alternative disease modifying treatment? (Y/N)
Why does the patient take <13mg/kg/day of UDCA?<br>-Optimal dose not offered by clinician<br>-Optimal dose declined by patient<br>-Intolerance of optimum dose<br>-Unknown<br>-Others
Has the patient taken UDCA for more than 12 months? (Y/N)
Based on the latest investigations, is the patient at high risk of disease progression? (Y/N)<br>*(based on the locally used definition of inadequate UDCA response, e.g. ALP > 1.67 x ULN after at least 12 months treatment)*
Was the patient referred to SLT MDT for consideration of second-line therapy? (Y/N)
Does the patient take any other disease-modifying treatment of PBC? (Y/N)
Which other disease-modifying treatment does the patient take?<br>-Obeticholic acid<br>-Bezafibrate<br>-Fenofibrate

-Budesonide<br>-Others (please specify)
Was this treatment recommended by the SLT MDT? (Y/N)
Do the clinic letters indicate that fatigue has been assessed within the last 24 months? (Y/N)<br>Do the clinic letters indicate that pruritus has been assessed within the last 24 months? (Y/N)<br>Did the patient have pruritus? (Y/N)<br>Does the patient currently receive treatment for PBC-related pruritus? (Y/N)<br>What is the treatment?<br>Is it clearly documented why the patient is not treated with Pruritus?
What treatment does the patient currently receive for PBC-related pruritus?<br>-Anti-histamines<br>-Cholestyramine<br>-Rifampicin<br>-Naltrexone<br>-Gabapentin<br>-Sertraline<br>-Others (what is the treatment – free text)
Is it clearly documented why the patient is not treated for pruritus? (Y/N)
Has the patient's risk of osteoporotic fracture been assessed within the last five years? (Y/N)<br>*(All types of risk assessment (FRAX score, DEXA scan, etc) as well as patient age and other health factors should be considered).*
Does the patient have a clinically significant risk of fracture? (Y/N)<br>(*As informed by the FRAX score or DEXA scan)*
Was appropriate action taken to reduce the risk of osteoporotic fracture? (Y/N)
Does the patient have cirrhosis? (Y/N)<br>*(Based on recent biopsy, imaging, elastography or supportive laboratory findings)*
Has the patient had an USS of the liver in the last 6 months? (Y/N)
Is it clearly documented why the patient did not have an USS? (Y/N)
Is the reason COVID-19 related delay? (Y/N)
Does the patient have clinically significant portal hypertension? (Y/N)<br>*(Based on Baveno criteria, the Newcastle Varices Score or locally agreed criteria)*<br>Has the patient had an OGD within the last 3 years? (Y/N)
Is it clearly documented why the patient has not had an OGD within the last 3 years? (Y/N)<br>Is the reason COVID-19 related delay? (Y/N)
IS the latest serum bilirubin >50umol/L? (Y/N)<br>Was the patient discussed with an LT centre? (Y/N)
Did this discussion take place within 3 months of the bilirubin first reaching 50umol/L? (Y/N)
Are there any other features of decompensated cirrhosis? (Y/N)<br>*(consider: UKELD score, evidence of ascites, hepatic encephalopathy or variceal bleed)*
Was the patient discussed with a LT Centre? (Y/N)

Blood tests ***(most recently available)*****:**
-Serum bilirubin
-Serum ALP
-Serum ALT
-Serum AST
-Serum Albumin
-Platelet count

Table S4: Overall Summary of Audit Performance

Performance standard	Number of patients meeting audit standard/total number patients (%)	Target (%)
**Diagnosis**		
Patients fulfilling diagnosis of PBC	8937/8968 (99.7)	n/a
Abdominal ultrasound scan at baseline*	2194/2491 (88.1)	90%
Liver biopsy undertaken	2856/8937 (32.0)	n/a
Biopsy compatible with PBC	2538/2856 (88.9)	n/a
Patients with a local diagnosis of PBC/AIH overlap	679/8937 (7.6)	n/a
PBC/AIH overlap diagnosis supported by liver biopsy	508/679 (74.8)	90%
**First-Line Therapy**		
Patients receiving UDCA as first-line therapy	7864/8937 (88.0)	n/a
Reason clearly documented for those not receiving UDCA	721/998 (72.2)	n/a
Patients not receiving UDCA due to intolerance	362/721 (50.2)	n/a
Patients receiving UDCA as first line therapy or documented intolerance	8226/8937 (92.0)	90%
Patients receiving UDCA dose of at least 13mg/kg	4203/6053 (69.4)	90%

**Second-Line Therapy**		
UDCA-untreated patients receiving second-line therapy	206/998 (20.6)	n/a
Patients with inadequate UDCA response according to local centre thresholds	2102/7395 (28.4)	n/a
Patients with inadequate UDCA response receiving second-line therapy	1074/2102 (51.1)	90%
**Symptom Assessment**		
Assessment of fatigue within the last 24 months	5052/8937 (56.5)	90%
Assessment of pruritus within the last 24 months	5522/8937 (61.8)	90%
**Osteoporosis Fracture Risk Assessment**		
Osteoporosis fracture risk assessment within the last 5 years	4883/8937 (54.6)	80%
Appropriate action taken in patients found to have a clinically significant risk of fracture	1447/1566 (92.4)	n/a
**Transplant Discussion**		
Patients with bilirubin > 50 µmol/L or hepatic decompensation discussed with a transplant centre	222/443 (50.1)	90%
Patients, aged below 70 years, with bilirubin > 50 µmol/L or evidence of hepatic decompensation discussed with a transplant centre	166/259 (64.1)	90%
**Surveillance**		
6 monthly HCC surveillance in patients with cirrhosis	1399/1947 (71.9)	90%
Surveillance of gastroesophageal varices in patients with clinically significant portal hypertension	695/905 (76.8)	90%

Table S5: Summary of Audit Performance According to Nation

Performance standard	Number of patients meeting audit standard/total number patients (%)			
	England	Wales	Scotland	NI
**Diagnosis**				
Abdominal ultrasound scan at baseline*	1929/2205 (87.5)	41/44 (93.2)	212/227 (93.4)	12/15 (80.0)
Liver biopsy undertaken	2509/7690 (32.6)	83/237 (35.0)	245/953 (25.7)	19/57 (33.3)
Biopsy compatible with PBC	2222/2509 (88.6)	75/83 (90.4)	223/245 (91.0)	18/19 (94.7)
Patients with local diagnosis of PBC/AIH overlap	582/7690 (7.6)	24/237 (10.1)	68/953 (7.1)	5/57 (8.8)
PBC/AIH overlap diagnosis confirmed by liver biopsy	433/582 (74.4)	21/24 (87.5)	49/68 (72.1)	5/5 (100)
**First-Line Therapy**				
Patients receiving UDCA as first line therapy	6742/7690 (87.7)	212/237 (89.5)	861/953 (90.3)	49/57 (86.0)
Reason clearly documented in patients not receiving UDCA	639/874 (73.1)	11/25 (44.0)	63/91 (69.2)	8/8 (100)
Patients documented to be UDCA-intolerant	320/639 (50.1)	5/11 (45.5)	30/63 (47.6)	7/8 (87.5)
Patients receiving UDCA as first line therapy or documented to be intolerant	7062/7960 (88.7)	217/237 (91.5)	891/953 (93.5)	56/57 (98.2)
Patients receiving UDCA of at least 13mg/kg	3533/5011 (70.5)	130/185 (70.3)	522/832 (62.7)	18/25 (72.0)
**Second-Line Therapy**				
UDCA-untreated patients receiving second-line therapy	117/874 (13.4)	3/25 (12.0)	20/91 (22.0)	6/8 (75.0)

Patients with inadequate UDCA response according to local centre thresholds	1826/6317 (28.9)	62/207 (30.0)	198/823 (24.1)	17/48 (35.4)
Patients with inadequate UDCA response receiving second-line therapy	927/1825 (50.8)	31/62 (50.0)	103/198 (52.0)	13/17 (76.5)
**Symptom Assessment**				
Assessment of fatigue within the last 24 months	4349/7690 (56.6)	134/237 (56.5)	527/953 (55.3)	42/57 (73.7)
Assessment of pruritus within the last 24 months	4758/7690 (61.9)	154/237 (65.0)	570/953 (59.8)	40/57 (70.2)
**Osteoporosis Fracture Risk Assessment**				
Risk assessment for osteoporosis within the last 5 years	4216/7690 (54.8)	106/237 (44.7)	531/953 (55.7)	30/57 (52.6)
Appropriate action taken in patients found to have a clinically significant risk of fracture	1249/1362 (91.7)	25/28 (89.3)	167/170 (98.2)	6/6 (100)
**Transplant Discussion**				
Patients with elevated bilirubin > 50 µmol/L or evidence of decompensation discussed with transplant centre	196/380 (51.6)	2/17 (11.8)	24/46 (52.2)	0/0 (0.0)
Patients, aged below 70, with elevated bilirubin > 50 µmol/L or evidence of decompensation discussed with transplant centre	143/216 (66.2)	2/11 (18.0)	21/32 (65.6)	0/0 (0.0)
**Surveillance**				
6 monthly US surveillance in patients with cirrhosis	1152/1639 (70.3)	53/72 (73.6)	184/224 (82.1)	10/12 (83.3)
Surveillance of gastroesophageal varices in patients with clinically significant portal hypertension	590/748 (78.9)	17/22 (77.3)	83/130 (63.8)	5/5 (100.0)

Table S6: Comparison of Audit Performance Between Specialist and Non-Specialist Centres

Performance standard	Number of patients meeting standard/total number patients (%)		OR	p-value†	95% CI Lower	95% CI Upper
	Specialist centres	Non-Specialist centres				
**Diagnosis**						
Abdominal ultrasound scan at baseline*	811/951 (85.3)	1118/1254 (89.2)	0.71	0.012	0.54	0.93
Liver biopsy undertaken	1410/3902 (36.1)	1099/3788 (29.0)	1.38	<0.001	1.26	1.53
Biopsy compatible with PBC	1276/1410 (90.5)	946/1099 (86.1)	1.54	<0.001	1.19	1.95
Patients with a local diagnosis of PBC/AIH overlap	277/3902 (7.1)	305/3787 (8.1)	0.87	0.121	0.73	1.04
PBC/AIH overlap diagnosis supported by liver biopsy	225/277 (81.2)	208/305 (68.2)	2.00	<0.001	1.35	3.03
**First-Line Therapy**						
Patients receiving UDCA as first line therapy	3466/3902 (88.8)	3276/3788 (86.5)	1.20	0.002	1.08	1.43
Reason clearly documented for those not receiving UDCA	327/414 (79.0)	312/460 (67.8)	1.78	<0.001	1.28	2.46
Patients not receiving UDCA due to intolerance	193/327 (59.0)	127/312 (40.7)	2.10	<0.001	1.51	2.91
Patients receiving UDCA as first line therapy or documented to be intolerant	3659/3902 (93.8)	3403/3788 (89.8)	1.70	<0.001	1.44	2.02
Patients receiving UDCA dose of at least 13mg/kg	1980/2664 (74.3)	1553/2347 (66.2)	1.48	<0.001	1.31	1.68
**Second-Line Therapy**						

UDCA-untreated patients receiving second-line therapy	106/414 (25.6)	71/460 (15.4)	1.88	<0.001	1.33	2.68
Patients with inadequate UDCA response according to local centre thresholds	1053/3298 (31.9)	772/3019 (25.6)	1.36	<0.001	1.22	1.52
Patients with inadequate UDCA response receiving second-line therapy	699/1053 (66.4)	228/772 (29.6)	4.69	<0.001	3.82	5.76
**Symptom Assessment**						
Assessment of fatigue within the last 24 months	2212/3902 (56.7)	2137/3788 (56.4)	1.01	0.82	0.92	1.11
Assessment of pruritus within the last 24 months	2547/3902 (65.3)	2211/3788 (58.4)	1.34	<0.001	1.22	1.47
**Osteoporosis Fracture Risk Assessment**						
Osteoporosis fracture risk assessment within the last 5 years	2343/3902 (60.0)	1873/3788 (49.4)	1.53	<0.001	1.40	1.68
Appropriate action taken in patients found to have a clinically significant risk of fracture	641/698 (91.8)	608/664 (91.6)	1.04	0.92	0.69	1.55
**Transplant Discussion**						
Patients with bilirubin > 50 µmol/L or hepatic decompensation discussed with a transplant centre	118/188 (62.8)	78/192 (40.6)	2.46	<0.001	1.60	3.80
Patients, aged below 70 years, with bilirubin > 50 µmol/L or hepatic decompensation discussed with a transplant centre	83/109 (76.1)	60/107 (56.1)	2.49	0.002	1.34	4.69
**Surveillance**						
6 monthly HCC surveillance in patients with cirrhosis	596/825 (72.2)	556/814 (68.3)	1.80	<0.001	1.26	2.58
	284/366 (77.6)	306/382 (80.1)	0.86	0.42	0.60	1.24

Surveillance of gastroesophageal varices in patients with clinically significant portal hypertension						

†Fisher's exact test
*Due to varying access of historical radiology, this analysis was only performed on patients diagnosed on/after 1st January 2017

Table S7: Summary of Audit Performance Across Regions in England

Performance standard	Number of patients meeting audit standard/total number patients (%)								
	North East	North West	Yorkshire	West Midlands	East Midlands	East of England	London	South East	South West
**Diagnosis**									
Abdominal ultrasound scan at baseline*	120/127 (94.5)	191/217 (88.0)	196/213 (92.0)	196/220 (89.0)	146/178 (82.0)	311/354 (87.9)	283/311 (91.0)	321/387 (82.9)	165/198 (83.3)
Liver biopsy undertaken	172/513 (33.5)	233/792 (29.4)	264/814 (32.4)	261/886 (29.5)	194/602 (32.2)	292/1022 (28.6)	470/1096 (42.9)	413/1153 (35.8)	210/812 (25.9)
Biopsy compatible with PBC	161/172 (93.6)	197/233 (84.6)	242/264 (91.7)	215/261 (82.4)	167/194 (86.1)	265/292 (90.8)	406/470 (86.4)	413/413 (100.0)	190/210 (90.5)
Patients with a local diagnosis of PBC/AIH overlap	37/513 (7.2)	54/792 (6.8)	76/814 (9.3)	51/886 (5.8)	44/602 (7.3)	55/1022 (5.4)	87/1096 (7.9)	111/1153 (9.6)	67/812 (8.2)
PBC/AIH overlap diagnosis supported by liver biopsy	28/37 (75.7)	38/54 (70.3)	65/76 (85.5)	27/51 (52.9)	34/44 (77.3)	38/55 (69.1)	63/87 (72.4)	85/111 (76.6)	55/67 (82.1)

**First-Line Therapy**									
Patients receiving UDCA as first-line therapy	465/513 (90.6)	696/792 (87.8)	742/814 (91.2)	760/886 (85.7)	512/602 (85.0)	911/1022 (89.1)	970/1096 (88.5)	1004/1153 (87.0)	682/812 (84.0)
Reason clearly documented in patients not receiving UDCA	40/47 (85.1)	68/84 (80.9)	57/68 (83.8)	67/112 (59.8)	68/88 (77.3)	83/109 (76.1)	98/121 (81.0)	74/134 (55.2)	84/111 (75.6)
Patients documented as being UDCA-intolerant	23/40 (57.5)	38/68 (55.9)	35/57 (61.4)	39/67 (58.2)	26/68 (38.2)	29/83 (34.9)	56/98 (57.1)	38/74 (51.3)	36/84 (42.9)
Patient receiving UDCA as first-line therapy or documented to be intolerant	488/513 (95.1)	734/792 (92.6)	777/814 (95.4)	799/886 (90.2)	538/602 (89.4)	940/1022 (91.9)	1026/1096 (93.6)	1042/1153 (90.4)	718/812 (88.4)
Taking UDCA at dose of at least 13mg/kg	282/375 (75.2)	313/464 (67.4)	391/574 (68.1)	443/633 (70.0)	148/256 (57.8)	536/735 (72.9)	526/738 (71.3)	623/843 (73.9)	271/393 (69.0)
**Second-Line Therapy**									
UDCA-untreated patients receiving second-line therapy	12/47 (25.5)	24/84 (28.6)	10/68 (14.7)	24/112 (21.4)	13/88 (14.8)	17/109 (15.6)	43/121 (35.5)	18/111 (16.2)	16/134 (11.9)
Patients with inadequate UDCA response according to local centre thresholds	136/433 (31.4)	203/636 (31.9)	166/697 (23.8)	230/710 (32.4)	172/492 (34.9)	186/855 (21.7)	284/924 (30.7)	275/919 (29.9)	173/651 (26.6)

Patients with inadequate UDCA response receiving second-line therapy	84/136 (61.8)	84/203 (41.4)	65/166 (39.1)	137/230 (59.6)	93/172 (54.0)	114/186 (61.2)	153/284 (53.9)	121/275 (44.0)	76/173 (43.9)
**Symptom Assessment**									
Assessment of fatigue within the last 24 months	360/513 (70.1)	389/792 (49.1)	389/814 (47.8)	512/886 (57.8)	342/602 (56.8)	590/1022 (57.7)	657/1096 (59.9)	660/1153 (57.2)	450/812 (55.4)
Assessment of pruritus within the last 24 months	396/513 (77.2)	419/792 (52.9)	482/814 (59.2)	523/886 (59.0)	342/602 (56.8)	618/1022 (60.5)	779/1096 (71.1)	726/1153 (63.0)	473/812 (58.3)
**Osteoporosis Fracture Risk Assessment**									
Osteoporosis fracture risk assessment within the last 5 years	350/513 (68.2)	404/792 (51.0)	520/814 (63.9)	298/886 (33.6)	269/602 (44.7)	632/1022 (61.8)	628/1096 (57.3)	693/1153 (60.1)	422/812 (52.0)
Appropriate action taken in patients found to have a clinically significant risk of fracture	92/106 (86.8)	119/134 (88.8)	142/154 (92.2)	65/71 (91.5)	72/75 (96.0)	148/169 (87.6)	222/240 (92.5)	238/251 (94.8)	151/162 (93.2)
**Transplant Discussion**									
Patients with bilirubin > 50 µmol/L or hepatic decompensation discussed with a transplant centre	7/12 (50.0)	8/34 (23.5)	19/26 (73.0)	63/81 (77.7)	9/31 (29.0)	32/54 (59.2)	33/49 (61.2)	15/63 (23.8)	10/30 (33.3)

Patients, aged below 70 years, with bilirubin > 50 µmol/L or hepatic decompensation discussed with a transplant centre	4/4 (100)	7/23 (30.4)	17/20 (85.0)	51/56 (91.1)	6/13 (46.1)	24/31 (77.4)	17/26 (65.4)	10/28 (35.7)	9/16 (56.3)
**Surveillance**									
6 monthly HCC surveillance in patients with cirrhosis	74/90 (82.2)	113/169 (66.8)	106/147 (72.1)	176/249 (70.7)	71/110 (64.5)	166/243 (68.3)	194/272 (71.3)	141/215 (65.6)	111/144 (77.1)
Surveillance of gastroesophageal varices in patients with clinically significant portal hypertension	23/33 (69.7)	47/62 (75.8)	82/101 (81.2)	98/122 (80.3)	29/46 (63.0)	83/97 85.6)	75/100 (75.0)	83/107 (77.6)	70/80 (87.5)

Fig. S1: Summary of Audit Performance Across Regions in England

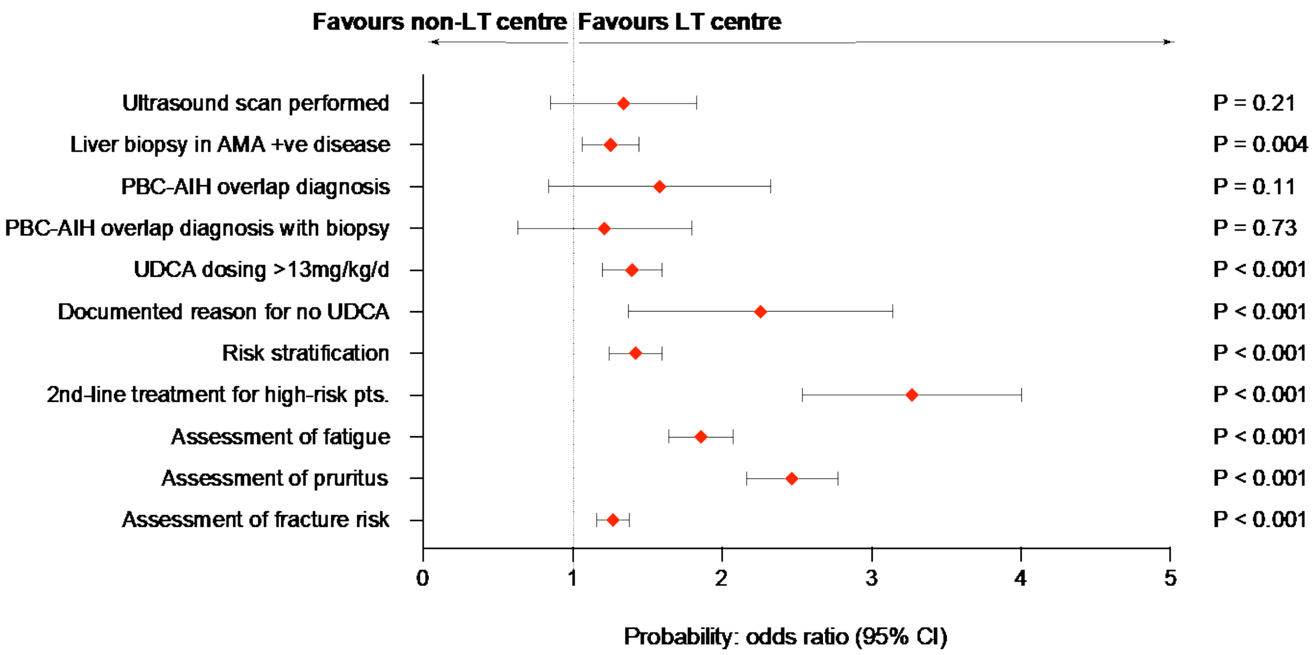

Forrest plot indicating the probability of a centre meeting a particular audit standard according to status as a liver transplant versus non-transplant unit.
