# Supplementary material for: Critical shortfalls in the management of PBC: Results of a UK-wide, population-based evaluation of care delivery
Source: JHEP Rep. 2023 Oct 16;6(1):100931. doi: 10.1016/j.jhepr.2023.100931 (PMC10711474; doi:10.1016/j.jhepr.2023.100931)
Supplement: Multimedia component 4 [file mmc4.pdf]

Critical shortfalls in the management of PBC: Results of a UK-wide, population-based evaluation of care delivery

Nadir Abbas,[1,2,3,†] **Rachel Smith**,[4,5,†] Steven Flack,[4] Vikram Bains,[5] Richard J. Aspinall,[6] Rebecca L. Jones,[7] Laura Burke,[7] Douglas Thorburn,[8] Michael Heneghan,[9] Andrew Yeoman,[10] Joanna Leithead,[11] Conor Braniff,[12] Andrew Robertson,[13] Chris Mitchell,[14] Collette Thain,[14] Robert Mitchell-Thain,[15] David Jones,[16,17] Palak J. Trivedi,[1,2,3,18,*,‡] George F. Mells,[4,5,*,‡] Laith Alrubaiy [19,20,*,‡], on behalf of the UK-PBC audit group

[1]National Institute for Health and Care Research (NIHR) Birmingham Biomedical Research Centre (BRC), Centre for Liver and Gastrointestinal Research, University of Birmingham, Birmingham, UK; [2]Liver Unit, University Hospitals Birmingham Queen Elizabeth, Birmingham, UK; [3]Institute of Immunology and Immunotherapy, University of Birmingham, Birmingham, UK; [4]Academic Department of Medical Genetics, University of Cambridge, Cambridge, UK; [5]Cambridge Liver Unit, Cambridge University Hospitals NHS Foundation Trust, Cambridge, UK; [6]Department of Gastroenterology and Hepatology, Portsmouth Hospitals University NHS Trust, Queen Alexandra Hospital, Portsmouth, UK; [7]Department of Hepatology, Leeds Teaching Hospitals NHS Trust, Leeds, UK; [8]Department of Hepatology and Liver Transplantation, Royal Free Hospital NHS Foundation Trust, London, UK; [9]Institute of Liver Studies, King's College Hospital NHS Foundation Trust, Denmark Hill, London, UK; [10]Aneurin Bevan University Health Board, Newport, UK; [11]Department of Hepatology, Forth Valley Royal Hospital, Larbert, UK; [12]Department of Hepatology, Regional Liver Unit, Belfast Health and Social Care Trust, Belfast, UK; [13]Department of Hepatology, Glasgow Royal Infirmary, Glasgow, UK; [14]PBC Foundation, Edinburgh, UK; [15]Global Liver Institute, UK; [16]Liver Unit, Newcastle upon Tyne Hospitals NHS Foundation Trust, Newcastle, UK; [17]NIHR Newcastle Biomedical Research Centre, Newcastle University, Newcastle, UK; [18]Institute of Applied Health Research, University of Birmingham, Birmingham, UK; [19]Department of Gastroenterology, St Mark's Hospital and Academic Institute, London, UK; [20]Swansea University Medical School, Swansea, UK

JHEP Reports **2024.** https://doi.org/10.1016/j.jhepr.2023.100931

Background & Aims: Guidelines for the management of primary biliary cholangitis (PBC) were published by the British Society of Gastroenterology in 2018. In this study, we assessed adherence to these guidelines in the UK National Health Service (NHS).
Methods: All NHS acute trusts were invited to contribute data between 1 January 2021 and 31 March 2022, assessing clinical care delivered to patients with PBC in the UK.
Results: We obtained data for 8,968 patients with PBC and identified substantial gaps in care across all guideline domains. Ursodeoxycholic acid (UDCA) was used as first-line treatment in 88% of patients (n = 7,864) but was under-dosed in one-third (n = 1,964). Twenty percent of patients who were UDCA-untreated (202/998) and 50% of patients with inadequate UDCA response (1,074/2,102) received second-line treatment. More than one-third of patients were not assessed for fatigue (43%; n = 3,885) or pruritus (38%; n = 3,415) in the previous 2 years. Fifty percent of all patients with evidence of hepatic decompensation were discussed with a liver transplant centre (222/443). Appropriate use of second-line treatment and referral for liver transplantation was significantly better in specialist PBC treatment centres compared with non-specialist centres (p <0.001).
Conclusions: Poor adherence to guidelines exists across all domains of PBC care in the NHS. Although specialist PBC treatment centres had greater adherence to guidelines, no single centre met all quality standards. Nationwide improvement in the delivery of PBC-related healthcare is required.
Impact and implications: This population-based evaluation of primary biliary cholangitis, spanning four nations of the UK, highlights critical shortfalls in care delivery when measured across all guideline domains. These include the use of liver biopsy in diagnosis; referral practice for second-line treatment and/or liver transplant assessment; and the evaluation of symptoms, extrahepatic manifestations, and complications of cirrhosis. The authors therefore propose implementation of a dedicated primary biliary cholangitis care bundle that aims to minimise heterogeneity in clinical practice and maximise adherence to key guideline standards.

© 2023 The Author(s). Published by Elsevier B.V. on behalf of European Association for the Study of the Liver (EASL). This is an open access article under the CC BY license (http://creativecommons.org/licenses/by/4.0/).

Introduction

An estimated 25,000 people in the UK live with primary biliary cholangitis (PBC),[1] a cholestatic liver disease which progresses to cirrhosis and its attendant complications in many patients. Although rare, PBC accounts for approximately one-tenth of liver transplant (LT) activity in the UK.[2] Progression to end-stage liver

Keywords: Adherence; Autoimmune liver disease; Bezafibrate; Fenofibrate; Guideline; Liver transplantation; Obeticholic acid; Second-line therapy; Service evaluation; Surveillance.
Received 28 July 2023; accepted 15 September 2023; available online 16 October 2023
† Joint first author.
‡ Joint senior author.
* Corresponding authors. Address: National Institute for Health Research (NIHR) Birmingham Liver Biomedical Research Centre, University of Birmingham College of Medical and Dental Sciences, Birmingham, Birmingham, UK (P. J. Trivedi); Department of Gastroenterology, St Mark's Hospital, London HA1 3UJ, UK (L. Alrubaiy); Academic Department of Medical Genetics, University of Cambridge, Cambridge, UK (G. F. Mells).
E-mail addresses: p.j.trivedi@bham.ac.uk (P.J. Trivedi), gfm26@cam.ac.uk (G.F. Mells), Laith.al-rubaiy@nhs.net (L. Alrubaiy).

disease and the need for transplantation can be mitigated by optimal use of disease-modifying therapies.[3–5]

In 2018, the British Society of Gastroenterology (BSG) updated its guidelines on the management of PBC. These guidelines describe three pillars of care: (1) 'Treat & Risk Stratify', emphasising the importance of optimally dosed first-line therapy, with timely initiation of second-line therapy (SLT) in patients with inadequate ursodeoxycholic acid (UDCA) response; (2) 'Stage & Survey', highlighting the value of surveillance for hepatocellular carcinoma (HCC) in patients with cirrhosis, screening for gastroesophageal varices in those with clinically significant portal hypertension, and prompt LT assessment for those with hepatic decompensation; and (3) 'Actively Manage', stressing the need to evaluate and treat symptoms such as pruritus, and associated conditions such as osteoporosis. Additionally, the BSG guidelines included several service standards that were intended to be a benchmark for PBC-related healthcare (Table 1).

We recently piloted an audit of PBC-related healthcare in 11 National Health Service (NHS) hospitals across the UK, which showed that none of the participating centres had achieved the BSG standards.[6] In the current study, recognising the failings were likely to be systemic, we extended our evaluation of PBC-related healthcare to NHS centres throughout the UK. We aimed to (1) evaluate overall performance against key service standards; (2) compare performance between specialist PBC treatment centres and non-specialist centres; and (3) compare prescribing rates for SLT across the constituent nations of the UK, which have adopted different models for SLT delivery.

Patients and methods

Selection of benchmark standards

We convened a working group in August 2020, consisting of hepatologists and gastroenterologists, patient representatives, and a data manager. To ensure adequate representation, we selected physicians from specialist and non-specialist centres across the four nations of the UK (Data S1). Following a consensus voting process, the working group agreed on the scope and standards of the audit, which were adopted from the service standards listed in the 2018 BSG PBC guidelines, the 2016 National Institute for Health and Care Excellence (NICE) guidelines on cirrhosis management, and the 2015 BSG guidelines on varices in cirrhosis[7] (Table S1). Comparison of these standards with international PBC guidelines published by the European Association for Study of the Liver (EASL) and other international bodies is provided in Table S2.

Site invitation and case finding strategy

All NHS acute trusts in the UK were invited to participate ([a]n acute trust is an organisational unit that provides secondary care services in the NHS). To maximise study participation, we established a national trainee network, consisting of junior doctors enrolled in specialty training (Data S1). Following registration of the audit, the local audit team (consisting of a consultant hepatologist or gastroenterologist and one or more specialty trainees) used active case-finding to identify patients with PBC under current follow-up. Active case-finding included one or more of the following strategies: (1) interrogation of hospital clinical coding databases (inpatient or outpatient) for individuals with an International Classification of Diseases (ICD)-10 code for PBC (K74.3), including those with concomitant codes for autoimmune hepatitis (AIH) (K73.2 and K75.4); (2) interrogation of immunology laboratory databases for patients with PBC-specific autoantibodies; (3) searching of histopathology laboratory databases for patients with liver biopsies compatible with PBC; and (4) screening of gastroenterology or hepatology departmental case notes and databases for patients with PBC, including those with features of AIH. For patients under follow-up in a local (non-specialist) centre but referred to a regional (specialist) centre for SLT, data were captured from the local centre to avoid duplication. Liver transplant recipients were excluded.

Data collection and quality control

We created an electronic case report form (eCRF) using the Research Electronic Data Capture platform (REDCap; Vanderbilt University, Nashville, TN, USA), a secure web-based application licensed by the University of Cambridge (Cambridge, UK). Data capture included the patient's sex, age group, and details about their PBC including diagnosis, first- and second-line treatment, risk of disease progression (defined as an abnormal bilirubin and/or alkaline phosphatase [ALP]>1.67 × upper limit of normal [ULN] after ≥12 months of treatment), symptom assessment, fracture risk assessment, HCC and varices surveillance, and whether a patient met referral criteria for transplant assessment. No patient identifiable details were collected. We provided audit teams with a user guide to support data entry and define data fields (Table S3). Following the submission of eCRFs, the data manager checked for omissions and resolved these with the local audit team. The period of data capture extended from 1 January 2021 until 31 March 2022. The data were subsequently downloaded from REDCap for analysis.

Data and statistical analysis

For each participating centre, we determined adherence to the audit standards. We then compared adherence according to type of centre (specialist *vs.* non-specialist centre), geographical region and model of SLT delivery, based on the following considerations unique to the UK.

Table 1. Summary of BSG Service Standards.

Service standard	Target
All patients with suspected PBC should have an abdominal ultrasound as part of their baseline assessment.	90%
All patients should be offered first-line treatment with UDCA at 13–15 mg/kg/day.	90%
Individualised risk stratification using biochemical response indices is recommended following 1 year of UDCA therapy.	80%
All patients should be evaluated for the presence of symptoms, in particular fatigue and pruritus.	90%
All patients with a bilirubin >50 μmol/L or evidence of decompensated liver disease should be discussed with a hepatologist linked to a transplant programme (within 3 months).	90%
All patients should have a risk assessment for osteoporosis (within the last 5 years).	80%
When overlap with autoimmune hepatitis is suspected, liver biopsy with expert clinicopathological assessment should be undertaken to support diagnosis.	90%

BSG, British Society of Gastroenterology; PBC, primary biliary cholangitis; UDCA, ursodeoxycholic acid.

The four constituent nations of the UK (England, Scotland, Wales, and Northern Ireland) have adopted distinct models for the delivery of SLT for PBC. In England, patients eligible for SLT are referred to a regional multi-disciplinary team (MDT), located in a specialist hepatobiliary centre ('specialist centre') that is networked to neighbouring, non-specialist hospitals (Fig. 1). The specialist centre is responsible for the approval of SLT and the prescription of obeticholic acid (OCA). In Wales and Northern Ireland, SLT is decided by a national MDT. This contrasts from Scotland, where SLT can be prescribed by any Hepatologist or Gastroenterologist, without input from an MDT.

There are seven LT-centres located in the UK: six in England, one in Scotland, and none in Wales or Northern Ireland. Patients eligible for LT in regions that do not contain an LT-centre must be referred to centres in other regions.

Continuous variables are represented by the median value and IQR. We used the non-parametric Mann–Whitney *U* test to look for differences between two discrete groups, and the Kruskal–Wallis test (with Dunn's *post-hoc* correction) for more than two groups. Categorical variables are represented by numbers and percentages (%). We used a X^2 or Fisher's exact test to identify differences between groups, and the odds ratios (ORs) with 95% CIs to quantify those differences. We considered a value of p <0.05 in a two-sided test to be statistically significant. Analyses were conducted using SPSS Statistics v24.0 (SPSS Inc., Chicago, IL, USA).

Patient and public involvement

The PBC Foundation (www.pbcfoundation.org.uk) and Global Liver Institute (GLI) (globalliver.org) provided patient and public involvement, nominating two members and one member, respectively, to be on the working group. Patient representatives were involved in all aspects of the project, including the selection of audit standards, design of the eCRF, interpretation of the data, and writing of the manuscript.

Ethics and governance

The study was a service evaluation. No identifiable patient information was collected. Day-to-day management of individual patients was not affected. The study was registered with the hospital audit office of each participating site before data collection. The NHS code of confidentiality was followed by all sites.[8]

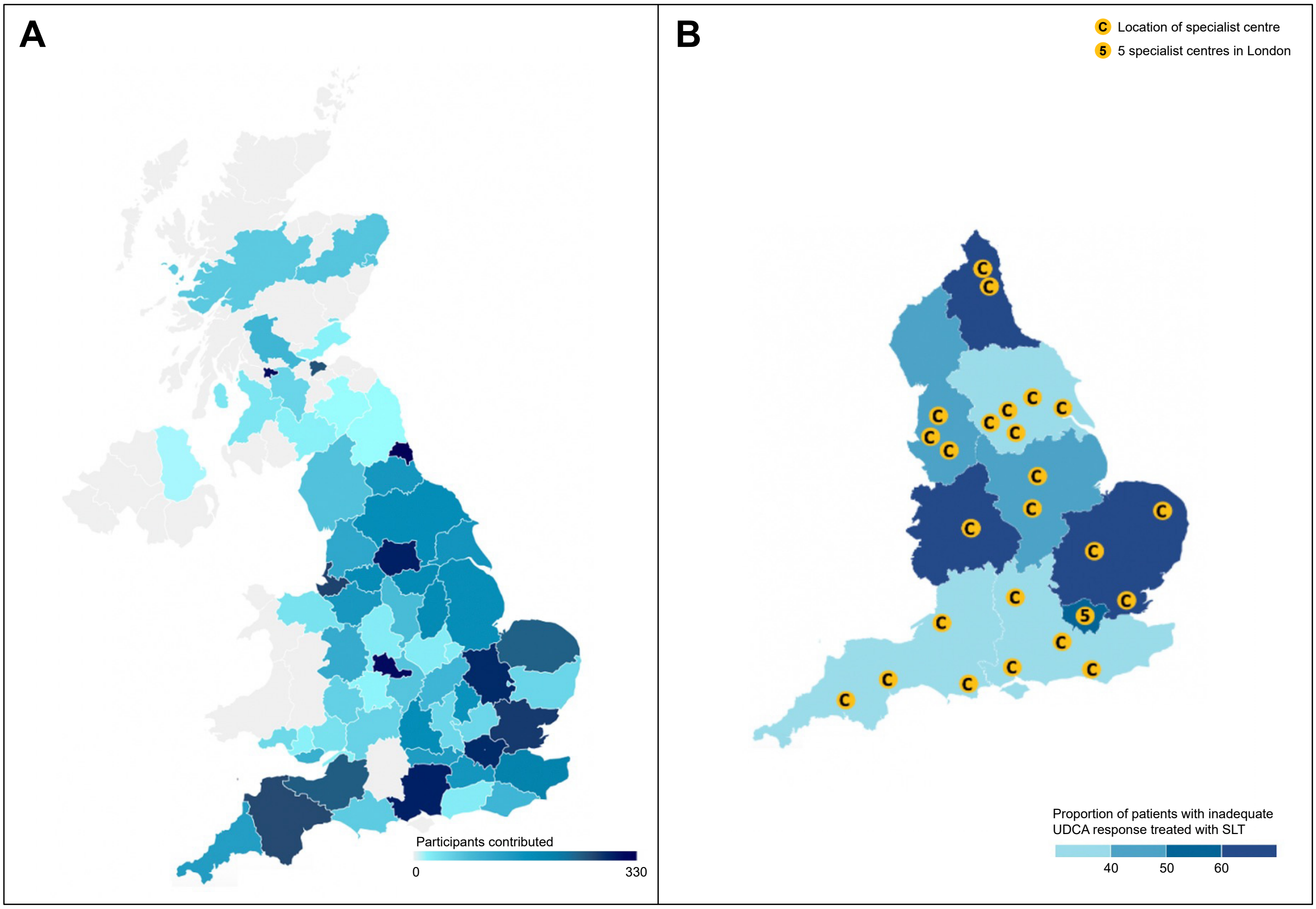

Fig. 1. Regions contributing to the PBC audit and location of specialist centres in England. Choropleth map indicating (A) the number of participants contributed by region, and (B) the location of regional specialist centres in England responsible for prescribing second-line treatment. SLT, second-line therapy; UDCA, ursodeoxycholic acid.

Results

Characteristics of the study population

We gathered data on 8,968 patients with PBC who were under follow up in 122 NHS centres across the UK. Most patients were women aged ≥50 years (n = 7,085; 79%). Eighty-one percent of patients (n = 7,263) were followed-up in a hepatology clinic; the remainder by gastroenterology (Table 2).

Liver biopsy

Almost one-third of patients underwent a liver biopsy (n = 2,856; 32%); of these, 68% (n = 1,945) had cholestatic biochemistry and either positive anti-mitochondrial autoantibodies (AMAs) and/or PBC-specific anti-nuclear autoantibodies (ANAs). As the use of liver biopsy may have declined since the release of EASL guidelines in 2017 and BSG guidelines in 2018, we compared rates before and after 2017: in all, 35% of patients diagnosed with PBC before 2017 had undergone a liver biopsy (2,239 of 6,446 patients), compared with 25% diagnosed since (617 of 2,491 patients) (p <0.001). Conversely, one in four patients reported to have PBC/AIH-overlap syndrome (n = 508) had not undergone histological evaluation (Fig. 2 and Table S4).

Disease-modifying treatment

Almost 90% of patients were treated with UDCA (n = 7,864), with patient weight and dose of UDCA available for n = 6,053. Nearly one-third of patients (n = 1,850) received a sub-optimal dose (<13 mg/kg/day), of whom 48% had an ALP value above the ULN (and 13%, an ALP >1.67 × ULN). In patients who were not treated with UDCA (n = 998), the reason was clearly documented in 72% (n = 721), the most common being drug intolerance (n = 362). Only one in five UDCA-untreated patients were prescribed an alternative second-line agent (n = 202). Amongst patients on UDCA monotherapy for at least 12 months, 2,102 had evidence of inadequate UDCA response; only half were prescribed SLT (n = 1,074; 51%). The choice of SLT therapy was split equally between OCA (n = 572) and fibric acid derivatives (bezafibrate or fenofibrate; n=571), with a small proportion of patients receiving both (n = 83), and a minority being treated with an alternative agent (n = 68). In England, Wales, and Northern Ireland, 73% of patients (682/933) with inadequate UDCA response who had not received SLT, had not been referred to the regional MDT. Sixty percent of these patients were under the age of 70 years.

Table 2. Characteristics of audit patients.

Patient characteristics	n (%)
Total patients	8,937
Female	7,941 (88.9)
Male	996 (11.1)
Age group (years)	
20–29	26 (0.3)
30–39	163 (1.8)
40–49	732 (8.2)
50–59	1,969 (22.0)
60–69	2,534 (28.4)
70–79	2,545 (28.5)
>80	968 (10.8)
Autoantibodies	
AMA	7,518 (84.1)
PBC-specific ANA	1,459 (16.3)
PBC/AIH-overlap	679 (7.6)
Clinic	
Hepatology	7,263 (81.3)
General gastroenterology	1,674 (18.7)
Nation	
England	7,690 (86.0)
Northern Ireland	57 (0.6)
Scotland	953 (10.7)
Wales	237 (2.7)

AIH, autoimmune hepatitis; AMA, anti-mitochondrial autoantibody; ANA, anti-nuclear autoantibody; PBC, primary biliary cholangitis.

Assessment of symptoms and extrahepatic manifestations

More than one-third of patients had not been assessed for fatigue (n = 3,885; 43%) or pruritus (n = 3,415; 38%) in the previous 24 months. Of those reported to have pruritus (n = 1,895), 67% received treatment, most often colestyramine (41%), antihistamines (30%), or rifampicin (17%). Only 55% of patients had undergone fracture risk assessment in the previous 5 years (n = 4,883). One-third of those assessed were deemed to have a clinically significant risk of fracture (n = 1,566; 32%), of whom 92% had received appropriate therapy to reduce this risk. Most patients who had not undergone fracture risk assessment were women above the age of 50 years (n = 2,596; 75%).

Discussion with a liver transplant centre

At the time of data collection, 443 patients had a serum total bilirubin >50 μmol/L or other features of decompensated cirrhosis; 50% of these patients had not been discussed with a transplant centre. Taking age >70 years to be an arbitrary exclusion criterion for LT, 36% of patients with hepatic decompensation (93/259) had not been referred for transplant assessment despite the advanced nature of their liver disease.

Surveillance for HCC and gastroesophageal varices

Overall, 1,947 patients were reported to have cirrhosis. Of these, 28% (n = 548) had not undergone ultrasound surveillance for HCC in the previous 6 months. In total, 905 patients were reported to have clinically significant portal hypertension, of whom 23% (n = 210) had not undergone endoscopic variceal surveillance in the previous 3 years. There was no clear documentation to account for the delay in ultrasound and endoscopic surveillance in 64% (n = 348) and 48% (n = 100) of patients, respectively. The COVID-19 pandemic was reported to account for the respective delays in just 8% and 5% of patients.

Variation in PBC-related healthcare across the UK

We then compared prescribing rates for SLT across the constituent nations. As very few patients had received SLT in Northern Ireland, data from this nation were excluded from the analysis. Despite different models of SLT delivery between nations, there were no differences in the proportion of eligible patients prescribed SLT in England (51%), Scotland (52%) or Wales (50%) (Table S5). There was, however, a difference in choice of therapy. In England and Wales, OCA was prescribed to 55% and 54% of eligible patients, respectively, whereas in Scotland, OCA was prescribed in only 16% eligible patients (p <0.001); the remainder received fibric acid derivatives.

In England, eligible patients were more likely to receive SLT if they were followed up in a specialist *vs.* a non-specialised centre (67% *vs.* 30%; OR 4.69, 95% CI 3.82–5.76; p <0.001) (Table S6 and Fig. S1). This was also evident in Scotland, where eligible patients were more likely to receive SLT if they were followed up in larger teaching hospitals compared with smaller district general hospitals (63% *vs.* 31%, OR 3.89, 95% CI 2.01–7.78; p <0.001) (Fig. 3). Specialist centres were also more likely to ensure UDCA was optimally dosed (74% *vs.* 66%; OR 1.48, 95% CI 1.31–1.68; p <0.001); ensure patients with cirrhosis underwent HCC

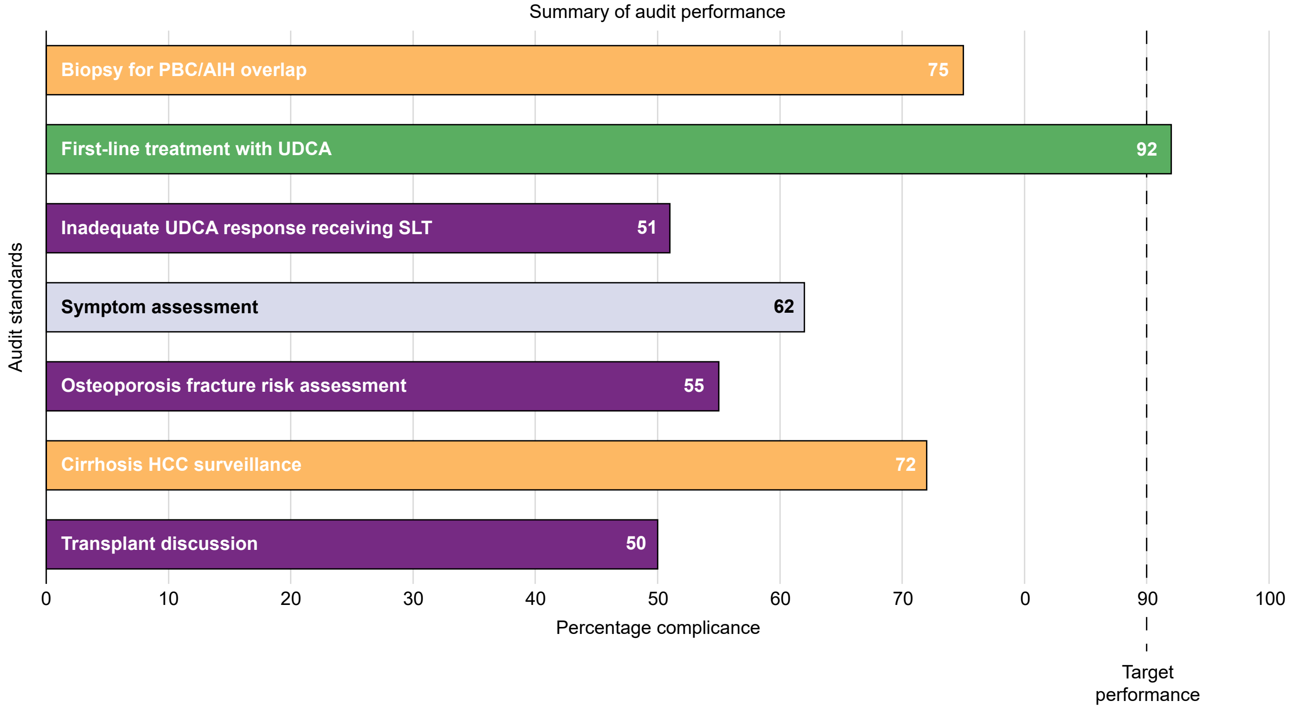

Fig. 2. Summary of overall audit performance. This histogram illustrates overall performance for each audit standard under evaluation across the four nations of the UK. AIH, autoimmune hepatitis; HCC, hepatocellular carcinoma; PBC, primary biliary cholangitis; SLT, second-line therapy; UDCA, ursodeoxycholic acid.

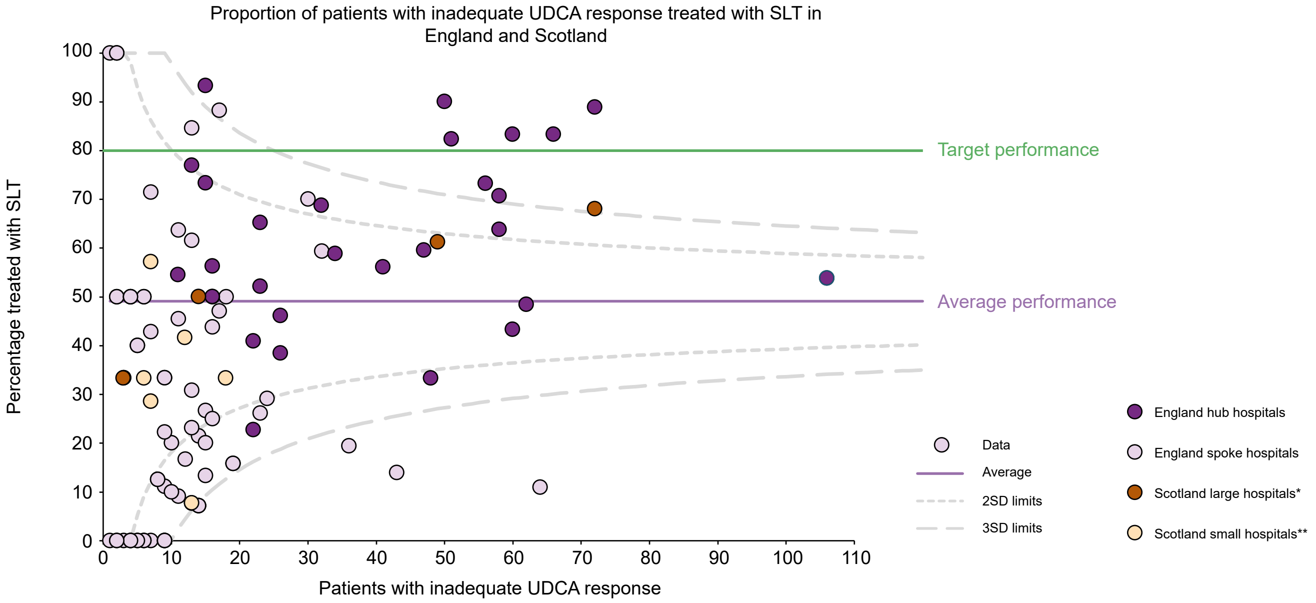

Fig. 3. Variation in the prescription of second-line treatment in England and Scotland. Funnel plot displaying the number of patients with inadequate response receiving second-line treatment according to the number of patients with inadequate UDCA response seen by each centre in England and Scotland. *Teaching hospital affiliated to a medical school. **District general hospital. SLT, second-line therapy; UDCA, ursodeoxycholic acid.

surveillance (72% *vs.* 68%; OR 1.80, 95% CI 1.26–2.58; p <0.001); and discuss patients with hepatic decompensation with an LT centre (76% *vs.* 56%, OR 2.49, 95% CI 1.34–4.69, p <0.001). They were more likely to assess pruritus (65% *vs.* 58%; OR 1.34, 95% CI 1.22–1.47; p <0.001) but no more likely to assess fatigue (57% *vs.* 56%; OR 1.01, 95% CI 0.92–1.11, p = 0.82).

We also analysed referral rates for LT in England, Scotland, and Wales. Northern Ireland recorded no cases of hepatic

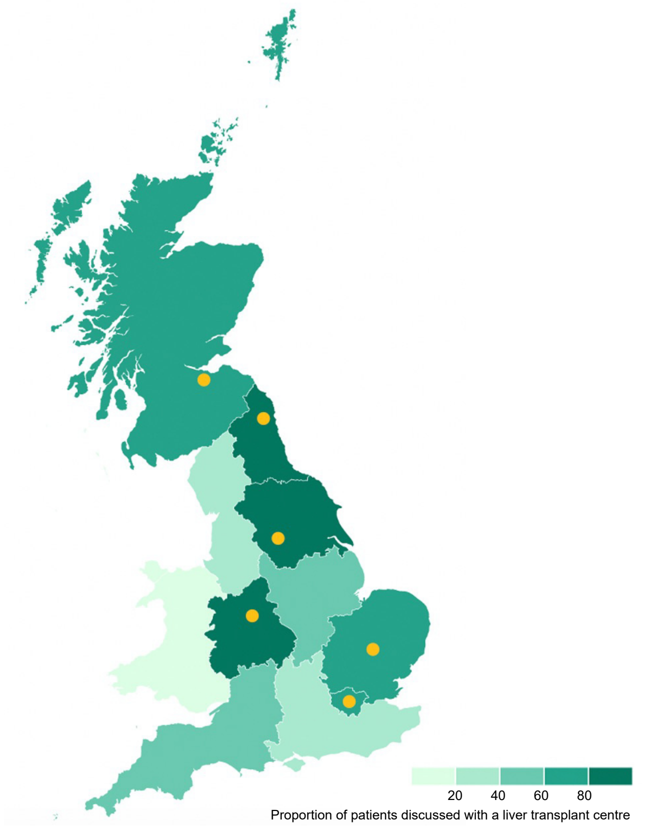

Fig. 4. Variation in referral for transplant assessment across three of the four nations in the UK. Choropleth map indicates the proportion of patients in each geographical region referred for liver transplant assessment. Transplant centres highlighted in yellow: from North to South indicate Edinburgh (the only liver transplant unit in Scotland), Newcastle, Leeds, Birmingham, Cambridge and London (note: two liver transplant centres are located in London, the Royal Free Hospital and King's College Hospital). No liver transplant centres are located in Wales.

decompensation during the service evaluation period so was not included. Patients eligible for LT were eight times more likely to be referred for transplant assessment if they lived in England or Scotland compared with Wales (52% *vs.* 11%; OR 7.98, 95% CI 1.82–72.6; p = 0.001). In England, patients eligible for LT were sevenfold more likely to be referred for LT if they lived in a region containing a LT-centre compared with regions not containing a LT-centre (82% *vs.* 40%; OR 6.99, 95% CI 3.60–13.90; p <0.001) (Fig. 4 and Table S7).

Discussion

PBC is a disease with a clinical and societal burden disproportionate to its prevalence. Although effective medical therapy exists, delays in diagnosis and treatment perpetuate poor outcomes.[9] Therefore, identifying deficiencies in healthcare has practical implications, and a first step in quality assurance for any clinical service.[10,11] In this UK-wide evaluation of PBC healthcare, we identified inadequate adherence to guidelines in all participating centres. Performance was suboptimal in all but one domain. Most striking was the proportion of eligible patients who were not receiving SLT, especially in non-specialist centres. Also striking was the proportion of patients with decompensated cirrhosis who were not referred for LT, particularly in regions without an LT centre.

In all, 50% of patients with inadequate UDCA response had not received SLT. In England, Wales, and Northern Ireland, nearly three-quarters of these patients had not been referred to an MDT, suggesting that the underlying problem is a failure to recognise when SLT is needed. Given that 60% of SLT-eligible but untreated patients were under the age of 70 years, it is unlikely that local centres had considered SLT for patients but deemed them unlikely to benefit owing to life-limiting comorbidity. Moreover, at the time of audit, OCA had only recently been approved by NICE for 3 years, so it is debatable whether non-specialist clinicians would have been best placed to decide against the use of a drug they had never prescribed. In Scotland, half of patients with inadequate UDCA response received SLT despite no requirement for MDT approval, supporting the failure to recognise patients in need of SLT, rather than the MDT, as a barrier to access. Addressing the recognition of patients eligible for SLT is a critical topic for future work.[12,13]

The probability of referral for LT was lowest amongst geographical regions lacking a transplant centre, suggesting that the national provision of such services is inequitable in terms of access across the UK. This finding mirrors previous observations, wherein serum bilirubin was greater in waitlisted patients with longer travel times to LT centres, consistent with delays in referral.[14] The issue of how best to enhance the equitable provision of LT services is challenging, but evidently improvements are needed. A regional, multi-disciplinary approach to the management of end-stage liver disease, LT outreach clinics within large gastroenterology units (jointly run by transplant hepatologists and local gastroenterologists), and ease of communication between referring and LT centres all play a role.[15,16]

Symptoms predict global quality of life for people living with PBC.[17] To this effect, we found evaluation of symptoms to be inadequate, with lack of recent symptom assessment in over one-third of patients. These findings align with the PBC Foundation patient experience survey, which found that 40% of patients had not been asked about their symptoms during the previous 12 months.[18] In said survey, nearly half of patients who raised queries about fatigue and a quarter of patients who asked about pruritus, received no advice. The availability of newer, quality-of-life tools provides an opportunity to quantify patient symptoms in routine clinical practice,[19] and readily identify patients who may benefit from lifestyle modifications, pruritus treatment and clinical trial participation.[20,21]

The diagnosis of PBC can be made using blood tests alone, supported by clinical history and presenting symptoms.[22] As such, histological confirmation is not needed except when there is diagnostic doubt.[23] Overuse of biopsy among patients with classical PBC serology, coupled with underuse in the group being attributed a diagnostic label of PBC/AIH-overlap, can be a result of conflicting statements provided between different guideline documents. For instance, the BSG recommendations on use of liver biopsy state that 'in PBC a liver biopsy should be done in clinically atypical cases such as failure to respond to UDCA.'[24] The same guideline document also states that 'Biopsy "may" also be useful in overlap syndrome'. These statements differ from the BSG PBC guidelines, potentially causing confusion for clinicians. Moreover, strict adherence to the guidelines may lead to some patients undergoing unnecessary and invasive procedures, and consequently delay in initiation of SLT. In turn, lack of histological confirmation in cases of putative PBC/AIH-overlap

risks harm, as some patients will receive long-term immunosuppression, despite lack of therapeutic benefit.[25] In a similar vein, contemporary recommendations for the assessment and management of bone health are lacking in chronic liver disease,[26] which may have resulted in lack of fracture risk assessment. Ensuring that there is alignment between guidelines that have a broad, more general hepatology remit to those that are PBC-specific may improve adherence to standards and limit variations in clinical practice.

Specialist centres generally had better performance than non-specialist centres, suggesting that familiarity with PBC is important for guideline adherence. It should be noted, however, that specialist centres still demonstrated sub-optimal disease management; no single centre achieved target performance across all domains. Improvement is therefore required across-the-board. Care bundles, which list the essential components of management, have been shown to improve compliance with guidelines.[27] In the UK, use of the BSG/British Association for the Study of the Liver (BASL) Decompensated Cirrhosis Care Bundle improved standards of care in patients with decompensated cirrhosis within the first 24 h of hospital admission.[28] A PBC care bundle is a potential solution to improve the delivery of PBC-related healthcare in all centres. In non-specialist centres, familiarity with PBC could be improved through attendance at virtual SLT MDT meetings, providing an opportunity for specialist experience to be shared, and the local cohorting of patients into dedicated clinics. Alongside a care bundle, these changes could facilitate the nationwide improvement required in the management of PBC. The development and implementation of a PBC care bundle forms the second phase of this audit and will be followed by a re-audit of PBC care delivery in selected centres to evaluate the impact of such a bundle on compliance with standards.

A notable limitation of our study is that reasons for non-adherence to guidelines were not captured, and similarly, we did not explore reasons for non-referral to regional specialist PBC MDTs. As such, our study was intended to provide a broad overview of care and identify deficiencies for focused evaluation in future work. The findings of this study have been disseminated to respective centres for reflection, allowing them to identify key areas and adopt strategies at a local level to improve the shortfalls identified. The working group will now focus on implementation of a PBC care bundle followed by a re-evaluation of clinical practice, as part of the wider UK quality improvement drive in liver services.[29]

Abbreviations

AIH, Autoimmune hepatitis; ALP, Alkaline phosphatase; AMA, Anti-mitochondrial autoantibody; ANA, Anti-nuclear autoantibody; BASL, British association for the study of the liver; BSG, British society of gastroenterology; EASL, European association for study of the liver; eCRF, Electronic case report form; GLI, Global liver institute; HCC, Hepatocellular carcinoma; ICD, International classification of diseases; LT, Liver transplant; MDT, Multi-disciplinary team; NHS, National health service; NICE, National institute for health and care excellence; OCA, Obeticholic acid; ORs, Odds ratios; PBC, Primary biliary cholangitis; REDCap, Research electronic data capture platform; SLT, Second-line therapy; UDCA, Ursodeoxycholic acid; UK, United Kingdom; ULN, Upper limit of normal.

Financial support

PT and NA receive institutional support from Birmingham NIHR BRC (National Institute for Health and Care Research Biomedical Research Centre): This paper presents independent research supported by the Birmingham NIHR BRC based at the University Hospitals Birmingham National Health Service Foundation Trust and the University of Birmingham. The views expressed are those of the author(s) and not necessarily those of the National Health Service, the NIHR, or the Department of Health. This study was supported by an unrestricted grant provided by Guts UK.

Conflicts of interest

The authors declare no conflicts of interest.

Please refer to the accompanying ICMJE disclosure forms for further details.

Authors' contributions

Data curation, methodology, statistical analysis, writing the original draft: Lead: NA, RS. Conceptualisation, supporting formal analysis, funding acquisition, supervision and writing original draft: PJT, GFM, LA. Patient and public involvement: CT, RMT. Reviewing and supporting the draft: SF, VB, RJA, RLJ, LB, DT, MH, AY, KJ, CB, AR, CM, DJ.

Data availability statement

Aggregate data that support the findings of this study are available on request from any of the corresponding authors.

Supplementary data

Supplementary data to this article can be found online at https://doi.org/10.1016/j.jhepr.2023.100931.

References

Author names in bold designate shared co-first authorship

[1] Webb GJ, Ryan RP, Marshall TP, Hirschfield GM. The epidemiology of UK autoimmune liver disease varies with geographic latitude. Clin Gastroenterol Hepatol 2021;19:2587–2596.
[2] NHS Blood and Transplant. Annual report on liver transplantation. 2021; https://www.odt.nhs.uk/statistics-and-reports/organ-specific-reports/.
[3] Corpechot C, Carrat F, Bahr A, Chrétien Y, Poupon RE, Poupon R. The effect of ursodeoxycholic acid therapy on the natural course of primary biliary cirrhosis. Gastroenterology 2005;128:297–303.
[4] Carbone M, Mells GF, Pells G, Dawwas MF, Newton JL, Heneghan MA, et al. Sex and age are determinants of the clinical phenotype of primary biliary cirrhosis and response to ursodeoxycholic acid. Gastroenterology 2013;144:560–569. e7; quiz e13–4.
[5] Lammers WJ, van Buuren HR, Hirschfield GM, Janssen HLA, Invernizzi P, Mason AL, et al. Levels of alkaline phosphatase and bilirubin are surrogate end points of outcomes of patients with primary biliary cirrhosis: an international follow-up study. Gastroenterology 2014;147:1338–1349. e5; quiz e15.
[6] Burke L, Flack S, Jones R, Aspinall RJ, Thorburn D, Jones DE, et al. The national audit of primary biliary cholangitis (PBC) in the United Kingdom: defining the audit dataset and data collection system. Cureus 2022;14:e25609.
[7] National Institute for Health and Care Excellence. NICE guideline NG 50. Cirrhosis in over 16s: assessment and management. Manchester: NICE; 2016.
[8] Confidentiality: NHS Code of Practise. https://www.gov.uk/government/publications/confidentiality-nhs-code-of-practice. [Accessed 7 November 2003].
[9] Carbone M, Nardi A, Flack S, Carpino G, Varvarpoulou N, Gavrilla C, et al. Pretreatment prediction of response to ursodeoxycholic acid in primary biliary cholangitis: development and validation of the UDCA Response Score. Lancet Gastroenterol Hepatol 2018;3:626–634.
[10] Dyson JK, Wong LL, Bigirumurame T, Hirschfield GM, Kendrick S, Oo YH, et al. Inequity of care provision and outcome disparity in autoimmune hepatitis in the United Kingdom. Aliment Pharmacol Ther 2018;48:951–960.

[11] Gordon V, Adhikary R, Appleby V, Das D, Day J, Delahooke T, et al. Diagnosis, presentation and initial severity of autoimmune hepatitis (AIH) in patients attending 28 hospitals in the UK. Liver Int 2018;38:1686–1695.
[12] Neuberger J, Patel J, Caldwell H, Davies S, Hebditch V, Hollywood C, et al. Guidelines on the use of liver biopsy in clinical practice from the British society of gastroenterology, the royal College of radiologists and the royal College of pathology. Gut 2020;69:1382–1403.
[13] Hussain N, Trivedi PJ. The inconvenient truth of primary biliary cholangitis/autoimmune hepatitis overlap syndrome. Clin Liver Dis 2022;26:657–680.
[14] Webb GJ, Hodson J, Chauhan A, O'Grady J, Neuberger JM, Hirschfield GM, et al. Proximity to transplant center and outcome among liver transplant patients. Am J Transpl 2019;19:208–220.
[15] Zhang YR, Wang H, Zhou N, Zhang YD, Lin Y, Wu LY, et al. A multidisciplinary team approach to the treatment of liver cirrhosis. J Inflamm Res 2021;14:5443–5450.
[16] Hulley JL, Douglass A, White S, Masson S. PTH-150 benefits of a liver transplant outreach clinic: increased referrals and patient satisfaction. Gut 2013;62:A272.
[17] Dyson JK, Wilkinson N, Jopson L, Mells G, Bathgate A, Heneghan MA, et al. The inter-relationship of symptom severity and quality of life in 2055 patients with primary biliary cholangitis. Aliment Pharmacol Ther 2016;44:1039–1050.
[18] Mitchell C, Eddy A, Neuberger J, Mitchell-Thain R. P13 Patient experience of primary biliary cholangitis (PBC) symptom evaluation and management in clinical appointments and pathways compared to the British Society of Gastroenterology (BSG)/UK-PBC PBC treatment and management guidelines: results from PBC Foundation mobile app patient survey. Gut 2022;71:A39–A41.
[19] Alrubaiy L, Mells G, Flack S, Bosomworth H, Hutchings H, Williams J, et al. PBC-10: a short quality of life measure for clinical screening in primary biliary cholangitis. Aliment Pharmacol Ther 2019;50:1223–1231.
[20] Freer A, Williams F, Durman S, Hayden J, Trivedi PJ, Armstrong MJ. Home-based exercise in patients with refractory fatigue associated with primary biliary cholangitis: a protocol for the EXerCise Intervention in cholesTatic LivEr Disease (EXCITED) feasibility trial. BMJ Open Gastroenterol 2021;8:e000579.
[21] **Khanna A**, **Jopson L**, Howel D, Bryant A, Blamire A, Newton JL, et al. Rituximab is ineffective for treatment of fatigue in primary biliary cholangitis: a phase 2 randomized controlled trial. Hepatology 2019;70:1646–1657.
[22] Hirschfield GM, Dyson JK, Alexander GJM, Chapman MH, Collier J, Hübscher S, et al. The British Society of Gastroenterology/UK-PBC primary biliary cholangitis treatment and management guidelines. Gut 2018;67:1568–1594.
[23] European Association for the Study of the Liver. EASL Clinical Practice Guidelines: the diagnosis and management of patients with primary biliary cholangitis. J Hepatol 2017;67:145–172.
[24] Tanaka A, Hirohara J, Nakano T, Matsumoto K, Chazouillères O, Takikawa H, et al. Association of bezafibrate with transplant-free survival in patients with primary biliary cholangitis. J Hepatol 2021;75:565–571.
[25] Murillo Perez CF, Fisher H, Hiu S, Kareithi D, Adekunle F, Mayne T, et al. Greater transplant-free survival in patients receiving obeticholic acid for primary biliary cholangitis in a clinical trial setting compared to real-world external controls. Gastroenterology 2022;163:1630–1642.e3.
[26] Collier J, Ninkovic M, Compston J. Guidelines on the management of osteoporosis associated with chronic liver disease. Gut 2002;50(Suppl 1):i1–i9.
[27] Levy MM, Rhodes A, Phillips GS, Townsend SR, Schorr CA, Beale R, et al. Surviving Sepsis Campaign: association between performance metrics and outcomes in a 7.5-year study. Crit Care Med 2015;43:3–12.
[28] Trainee Collaborative for Research and Audit, Hepatology UK. The BSG/BASL bundle for patients admitted with decompensated chronic liver disease improves standard of care but utilisation is poor across the UK. Clin Med (Lond) 2022;22(Suppl4):33–34.
[29] Improving quality in liver services (IQILS). https://www.iqils.org. [Accessed 29 November 2022].

Journal of Hepatology, Volume *6*

Supplemental information

Critical shortfalls in the management of PBC: Results of a UK-wide, population-based evaluation of care delivery

Nadir Abbas, Rachel Smith, Steven Flack, Vikram Bains, Richard J. Aspinall, Rebecca L. Jones, Laura Burke, Douglas Thorburn, Michael Heneghan, Andrew Yeoman, Joanna Leithead, Conor Braniff, Andrew Robertson, Chris Mitchell, Collette Thain, Robert Mitchell-Thain, David Jones, Palak J. Trivedi, George F. Mells, Laith Alrubaiy, and on behalf of the UK-PBC audit group

Critical shortfalls in the management of PBC: Results of a UK-wide, population-based evaluation of care delivery

Nadir Abbas, Rachel Smith, Steven Flack, Vikram Bains, Richard J. Aspinall, Rebecca L. Jones, Laura Burke, Douglas Thorburn, Michael Heneghan, Andrew Yeoman, Joanna Leithead, Conor Braniff, Andrew Robertson, Chris Mitchell, Collette Thain, Robert Mitchell-Thain, David Jones, Palak J. Trivedi, George F. Mells, Laith Alrubaiy on behalf of the UK-PBC audit group

Table of contents

Supplementary Data S1……………………………………………………………2

Table S1……………………………………………………………………………8

Table S2……………………………………………………………………………9

Table S3…………………………………………………………………………… 12

Table S4…………………………………………………………………………… 15

Table S5…………………………………………………………………………… 17

Table S6…………………………………………………………………………… 19

Table S7…………………………………………………………………………… 22

Fig. S1………………………………………………………………………………26

Supplementary Data S1: Audit Working Group and Trainee Network

Specialist Centres

1. Dr Richard J Aspinall, Queen Alexandra Hospital, Portsmouth, England
2. Dr Conor Braniff, Royal Victoria Hospital, Belfast, Northern Ireland
3. Professor Michael Heneghan, King's College Hospital, London, England
4. Professor David Jones, Freeman Hospital, Newcastle, England
5. Dr Rebecca Jones, St James's University Hospital, Leeds, England
6. Dr George Mells, Addenbrooke's Hospital, Cambridge, England[*]
7. Dr Douglas Thorburn, Royal Free Hospital, London, England
8. Dr Palak Trivedi, Queen Elizabeth Hospital, Birmingham, England*
9. Dr Andrew Yeoman, Royal Gwent Hospital, Newport, Wales

Non-Specialist Centres

10. Dr Laith Alrubaiy, St Mark's Hospital, London, England[*]
11. Dr Joanna Leithead, Forth Valley Hospital, Larbert, Scotland

Patient Representatives

12. Robert Mitchell-Thain, Global Liver Institute
13. Chris Mitchell, PBC Foundation, Edinburgh, Scotland
14. Collette Thain, PBC Foundation, Edinburgh, Scotland

Data Manager

15. Steve Flack, University of Cambridge, Cambridge, England

---

[*] Project co-lead

Supplementary Data S1 Continued: Trainee Network

Nadir Abbas[1], Basma Ahmed Abdelsalam[2], Samad Abdul[3], Duaa Abduljabbar[4], Anwar Abusrewil[5], Lauren Adams[6], Mohamed Adam[7], Salman Ahmed[8], Jawad Ali[9], Robin Aldridge[10], Tanzil Al Imran[10], Lance Alleyne[11], Laith Alrubaiy[12], Sarah Al-Shakhshir[13], Ismaeel Al-Talib[14], Muhammad Nauman Amin[15], Tanmay Anand[16], Bradley Arms-Williams[17], Fahad Ashraf[18], Muteeb Ashraf[2], Usama Aslam[19], Huma Asmat[20], Zaw Myo Aung[21], Desson Au-Yeung[22], Rosewan Aydogan[23], Nauman Babar[24], Faisal Baig[25,26] , Vikram Bains[27], Opeyemi Bamidele[28], Arindam Banerjee[29], M Fahd Baqai[30], Ezzeldin Barakat[31], Alina Barcan[32], Kris Bennett[30], Mandeep Kaur Bhandal[33], Azad Bhushan[34], Karl Bonello[35], Daniel Bordom[36], Joshua Bower[37], Gary Bray[38], Paul Brennan[39], Rebecca Brown[2], Andrei Brunchi[34], Laura Burke[40], Karen Burns[41], Iona Campbell[24], Roisin Campbell[42], Andra Caracostea[43], M Carbonell[44], Zillah Cargill[45], Jennifer Cathcart[46], Manpreet Chahal[47], Gayatri Chakrabarty[48], Michelle Cheung[49], Anmol Chikhlia[16], Xin Yi Choon[50], Yooyun Chung[51], Jennifer Clancy[49], Emma Coleman[52], Christopher Cooper[53], Zoe Cox[54], Jo Coyle[37], Dominic Crocombe[49], Joanna Crombie[28], Lourdes Cumlat[54], Courtney Davies[16], Janet Dearden[29], Mollie Delaney[1], Bruce Dickson[55], Grace Dolman[29], Hannah Donnelly[56], Rebecca Dunn[2], Louise Eales[57], Jonathan Easaw[58], Mostafa El Gindy[18], Kathy Ellwood[59], Maged Elsaie[11], Ahmed El-Sayed[60], Louise Evans[61], Samantha Farrow[35], Helen Fellows[62], Matthew Foxton[8,44], Keith George[91], Andrew Gerges[63], Ben Giles[92], David Gomez[64], Stephanie Goodman[54], Akshaye Gopaul[16], Sarah Gormley[28], Mahmoud Gouda[43], Aarav Gupta-Kaistha[31], Sarah Guthrie[65], Richard Hackett[8], Andreas Hadjinicolaou[66], Neil Halliday[49], Basem Hamzah[2], Bisam-ul Haq[1], James Hassall[67], Omar Herman[68,69], Mathew Hewitt[17], Amy Hicks[70], Lorraine Hill[71], Htar Htar Hlaing[72], Anna Howard[40], Catherine Hsu[73], Muhammad Javaid Iqbal[63], Jumi Isibor[93], Asem Ismail[94], Jamal W Jamal[48], Joel James[74], Pernia Javid[2], Nasir Javed[31], Dhaarica Jeyanesan[43], Rhiannon Jones[17], Laura Jopson[75], M. Junaid Aleem[76], Yiannis Kallis[29], Sophia Kanwar[58], Syed Karim[38], Christopher Kelly[104], Muhammad Usman Khalid[63], Asif Khan[105], Aria Khani[106], Yun Kim[107], Jade King[49], Meera Kirby[50], Sara Koo[108], Shemin Kovammal[23], Victoria Kronsten[43], Ayo Lagundoye[109], Jonathan Lam[49], Wai Liam Lam[62], Brooke Layard[84], Angela Liaros[110], Thinzar Linn[111], Megan Lofthouse[112], Ruairi Lynch[24], Mohan Madayala[77], Aarani Mahalingam[36], Moawad Mahgoub[78], Abdul Manan[64], Vanessa Martin[79], Ben Masterman[80], Charmaine Matthews[81], Iana Martini[16,82], Maria-Emanuela Maxan[83], Nicola McCallion[84], Matthew McCormack[76], Sarah McDermott131, Hannah McDowell[85], Kate McHugh[86], Stephanie Michail[81], Neil Mistry[87], Dhanoop Mohandas[16], Sean Mole[30], Claudia Moore-Gillon[73], James Morgan[67], James Morgan[88], Sean Morris[1], Jennifer Murray[50], Sruthi Murthy[89], Ruhullah Nasiri[63], Muhammad Ahsan Nasim[31], Rooshi Nathwani[49], Edward Nicholson[89], Gaurav Nigam[90], Murtaza Nomani[1], Kirsty Nixon[116], Laura Ogle[5], Olaku Okeke[97], Olaolu Olabintan[113], Elaine Ong Ming San[114], Josh Orpen-Palmer[115], Michael Orrell[22], Glenda Osuoha[40], Christina Owen[28], Aashish Pandya[49], Ioannis Papamargaritis[117], Maurizio Parker[118], Suvey Perera[119], Alex Perry[120], Robert Perry[73], Rory Peters[22], Naw April Phaw[111], Abimbola Phillips[121], Anju Phoolchund[107], Lushen Pillay[82], Emily Platten[111], Keith Pohl[122], Lucia Possamai[73], Krithivasan Praman[66], Yash Prasad[123], Andrew Punton[124], Abdul Hameed Rahimi[95], Satyasheel Ramful[111], Naomi Ratcliff[49], Zaira Rehman[64], Christy Riggott[125], Andrew Robertson[115], Emma Robinson[39], Simon Rushbrook[79], Nafisa Salih[126], David Samuel[96], Emma Saunsbury[127], Jenny Scott[24], Jayshri Shah[23], Jessica Shearer[128], Abhishek Sheth[89], Harsimranjit Sidhu[73], Abigail Silcock[97,98], Sophie Sinclair[21], Prabhsimran Singh[99], Rohit Sinha[100], Belinda Smith[73], R Sood[94], Chrysoula Spyridou[30],Jennifer Steen[129], Kate Stephen[43], Jason Stevenson[101], Elena Sumanariu[23,130], Nikki Summers[102], Liz Sweeney[81,103], Huey Tan[30], Olivia Taylor[82], Jennifer Tham[115], Amy Thatcher[131], Helen Thompson Jones[61], Qamar Tufail[15], Varun Udayachandran[23], Vithushan Vakeeswarasarma[135], Jessiya Veliyankodan Parambil[90], Mathew

Vithayathil[73], Edvard Volcek[82], Anand Vyas[28], Katy Waddell[42], Gwilym Webb[95], Emily Westlake[49], Aaron Wetten[5], Helen White[93], James Willsmore[132,133], Jenna Wooding[134], John Wye[21], Irvin Yeoh[111], Andrew Yeoman[87], Faisal Zahedi[136], Shakir Zaman[84], Andreea Zotescu[23], Seema Zulfikar[23]

1. Queen Elizabeth Hospital, University Hospitals Birmingham NHS Foundation Trust
2. Royal Surrey County Hospital, Royal Surrey County Hospital NHS Foundation Trust
3. East Surrey Hospital, Surrey and Sussex Healthcare NHS Trust
4. Worcester Royal Hospital, Worcestershire Acute Hospitals NHS Trust
5. Freeman Hospital , Newcastle upon Tyne Hospitals NHS Foundation Trust
6. Leighton Hospital, Mid Cheshire Hospitals NHS Foundation Trust
7. Wrexham Maelor Hospital, Betsi Cadwaladr University Health Board
8. Chelsea & Westminster Hospital, Chelsea and Westminster Hospital NHS Foundation Trust
9. Watford General Hospital, West Hertfordshire Hospitals NHS Trust
10. Torbay Hospital, South Devon Healthcare NHS Foundation Trust
11. Leicester Royal Infirmary, University Hospitals of Leicester NHS Trust
12. Northwick Park Hospital, London North West Healthcare NHS Trust
13. Royal Shrewsbury Hospital, Shrewsbury and Telford Hospital NHS Trust
14. Walsall Manor Hospital, Walsall Healthcare NHS Trust
15. Kettering General Hospital, Kettering General Hospital NHS Foundation Trust
16. Lister Hospital, East and North Hertfordshire NHS Trust
17. University Hospital of Wales, Cardiff and Vale University Health Board
18. King's Mill Hospital, Sherwood Forest Hospitals NHS Foundation Trust
19. Ipswich Hospital, East Suffolk and North Essex NHS Foundation Trust
20. Bedford Hospital, Bedford Hospitals NHS Trust
21. Luton and Dunstable Hospital, Bedfordshire Hospitals NHS Foundation Trust
22. John Radcliffe Hospital, Oxford University Hospitals NHS Trust
23. William Harvey Hospital, East Kent Hospitals University NHS Foundation Trust
24. Royal Infirmary of Edinburgh, NHS Lothian
25. Lincoln County Hospital, United Lincolnshire Hospitals NHS Trust
26. Pilgrim Hospital, United Lincolnshire Hospitals NHS Trust
27. Addenbrooke's Hospital, Cambridge University Hospitals NHS Foundation Trust
28. Musgrove Park Hospital , Taunton and Somerset NHS Foundation Trust
29. Royal London Hospital, Barts Health NHS Trust
30. Derriford Hospital, Plymouth Hospitals NHS Trust
31. Royal Derby Hospital , University Hospitals of Derby and Burton NHS Foundation Trust
32. Royal Hampshire County Hospital, Hampshire Hospitals NHS Foundation Trust
33. Frimley Park Hospital, Frimley Health NHS Foundation Trust
34. North Devon District Hospital, Northern Devon Healthcare NHS Trust
35. Victoria Hospital, Kirkcaldy, NHS Fife
36. Broomfield Hospital, Mid Essex Hospital Services NHS Trust
37. George Eliot Hospital, George Eliot Hospital NHS Trust
38. Southend University Hospital, Southend University Hospital NHS Foundation Trust
39. Ninewells Hospital, NHS Tayside
40. St James's University Hospital, Leeds Teaching Hospitals NHS Trust
41. Royal Lancaster infirmary, University Hospitals of Morecambe Bay NHS Foundation Trust

42. University Hospital Hairmyres, NHS Lanarkshire
43. King's College Hospital, King's College Hospital NHS Foundation Trust
44. West Middlesex University Hospital, Chelsea and Westminster Hospital NHS Foundation Trust
45. Maidstone Hospital, Maidstone and Tunbridge Wells NHS Trust
46. Aberdeen Royal Infirmary, NHS Grampian
47. Royal Stoke University Hospital, University Hospitals of North Midlands NHS Trust
48. East Surrey Hospital, Surrey and Sussex Healthcare NHS Trust
49. Royal Free Hospital , Royal Free London NHS Foundation Trust
50. Queen's Hospital, Barking, Havering and Redbridge University Hospitals NHS Trust
51. St George's University Hospital, St George's University Hospitals NHS Foundation Trust
52. Princess Royal Hospital, Brighton and Sussex University Hospitals NHS Trust
53. University College London Hospital , University College London Hospitals NHS Foundation Trust
54. James Cook University Hospital, South Tees Hospitals NHS Foundation Trust
55. Raigmore Hospital, NHS Highland
56. Gloucestershire Royal Hospital , Gloucestershire Hospitals NHS Foundation Trust
57. Medway Maritime Hospital, Medway NHS Foundation Trust
58. Royal Blackburn Hospital, East Lancashire Hospitals NHS Trust
59. Scunthorpe General Hospital, Northern Lincolnshire and Goole NHS Foundation Trust
60. Northwick Park Hospital, London North West Healthcare NHS Trust
61. Singleton Hospital, Swansea Bay University Health Board
62. Hereford County Hospital, Wye Valley NHS Trust
63. Diana Princess of Wales Hospital, Northern Lincolnshire and Goole NHS Foundation Trust
64. New Cross Hospital, Royal Wolverhampton Hospitals NHS Trust
65. Royal Bolton Hospital, Bolton NHS Foundation Trust
66. Hinchingbrooke Hospital , North West Anglia NHS Foundation Trust
67. Northern General, Sheffield Teaching Hospitals NHS Foundation Trust
68. Stoke Mandeville Hospital, Buckinghamshire Healthcare NHS Trust
69. Wycombe Hospital, Buckinghamshire Healthcare NHS Trust
70. Hull Royal Infirmary, Hull University Teaching Hospitals
71. Royal Alexandra Hospital, NHS Greater Glasgow and Clyde
72. West Suffolk Hospital , West Suffolk NHS Foundation Trust
73. St Mary's Hospital, Imperial College Healthcare NHS Trust
74. Royal Cornwall Hospital, Royal Cornwall Hospitals NHS Trust
75. Hexham General Hospital, Northumbria Healthcare NHS Foundation Trust
76. Warwick General Hospital, South Warwickshire NHS Foundation Trust
77. Royal Preston Hospital, Lancashire Teaching Hospitals NHS Foundation Trust
78. Dumfries and Galloway Royal Infirmary, NHS Dumfries & Galloway
79. Norfolk and Norwich University Hospital, Norfolk and Norwich University Hospitals NHS Foundation Trust
80. Yeovil District Hospital , Yeovil District Hospital NHS Foundation Trust
81. Royal Liverpool Hospital, Liverpool University Hospitals NHS Foundation Trust
82. Princess Alexandra Hospital, Princess Alexandra Hospital NHS Trust
83. Croydon University Hospital, Croydon Health Services NHS Trust
84. Royal Victoria Hospital, Belfast Health and Social Care Trust

85. University Hospital Coventry, University Hospitals Coventry and Warwickshire NHS Trust
86. Queen Elizabeth Hospital, Gateshead Health NHS Foundation Trust
87. Royal Gwent Hospital, Aneurin Bevan University Health Board
88. Sandwell General Hospital , Sandwell and West Birmingham Hospitals NHS Trust
89. Queen's Medical Centre, Nottingham University Hospitals NHS Trust
90. Milton Keynes University Hospital, Milton Keynes Hospital NHS Foundation Trust
91. Torbay Hospital, South Devon Healthcare NHS Foundation Trust
92. Queen Alexandra Hospital, Portsmouth Hospitals NHS Trust
93. Salford Royal Hospital, Salford Royal NHS Foundation Trust
94. Russells Hall Hospital, Dudley Group of Hospitals NHS Trust
95. Peterborough City Hospital, North West Anglia NHS Foundation Trust
96. Royal Glamorgan Hospital, Cwm Taf Morgannwg University Health Board
97. University Hospital of North Durham, County Durham and Darlington NHS Foundation Trust
98. Bishop Auckland Hospital , County Durham and Darlington NHS Foundation Trust
99. Selby War Memorial Hospital, York Teaching Hospital NHS Foundation Trust
100. Sunderland Royal Hospital, South Tyneside and Sunderland NHS Foundation Trust
101. Leighton Hospital, Mid Cheshire Hospitals NHS Foundation Trust
102. University Hospital Aintree, Liverpool University Hospitals NHS Foundation Trust
103. Southport Hospital, Southport & Ormskirk Hospital NHS Trust
104. Forth Valley Royal Hospital, NHS Forth Valley
105. Huddersfield Royal Infirmary, Calderdale And Huddersfield NHS Foundation Trust
106. North Middlesex Hospital, North Middlesex University Hospital NHS Trust
107. University Hospital Southampton, University Hospital Southampton NHS Foundation Trust
108. Cumberland Infirmary, North Cumbria University Hospitals NHS Foundation Trust
109. Northampton General Hospital, Northampton General Hospital NHS Trust
110. Whiston Hospital , St Helens and Knowsley Teaching Hospitals NHS Trust
111. University Hospital of North Tees, North Tees and Hartlepool NHS Foundation Trust
112. Medway Maritime Hospital, Medway NHS Foundation Trust
113. Royal County Sussex Hospital, Brighton and Sussex University Hospitals NHS Trust
114. Pinderfields Hospital, Mid Yorkshire Hospitals NHS Trust
115. Gartnavel General Hospital, NHS Greater Glasgow and Clyde
116. Manchester Royal Infirmary, Manchester University NHS Foundation Trust
117. Arrowe Park Hospital, Wirral University Teaching Hospital NHS Foundation Trust
118. Kingston Hospital, Kingston Hospital NHS Foundation Trust
119. University Hospital Hairmyres, NHS Lanarkshire
120. Bristol Royal Infirmary, University Hospitals Bristol NHS Foundation Trust
121. James Paget Hospital , James Paget University Hospitals NHS Foundation Trust
122. Royal Devon and Exeter Hospital, Royal Devon and Exeter NHS Foundation Trust
123. Countess of Chester Hospital, Countess of Chester Hospital NHS Foundation Trust
124. Borders General Hospital, NHS Borders
125. Harrogate Hospital , Harrogate and District NHS Foundation Trust
126. Royal Berkshire Hospital, Royal Berkshire NHS Foundation Trust
127. Royal United Hospital Bath , Royal United Hospitals Bath NHS Foundation Trust
128. York Hospital, York Teaching Hospital NHS Foundation Trust
129. University Hospital Crosshouse, NHS Ayrshire & Arran
130. Kent and Canterbury Hospital , East Kent Hospitals University NHS Foundation Trust

131. Mount Vernon Hospital, Hillingdon Hospitals NHS Foundation Trust
132. Ealing Hospital, London North West Healthcare NHS Trust
133. Central Middlesex Hospital, London North West Healthcare NHS Trust
134. Royal Bournemouth Hospital, Royal Bournemouth and Christchurch Hospitals NHS Foundation Trust
135. Hemel Hempstead Hospital, West Hertfordshire Hospitals NHS Trust
136. Basildon University Hospital , Basildon and Thurrock University Hospitals NHS Foundation Trust

Table S1: UK-PBC Audit Standards

Audit Standards		Target Performance
1	All patients with suspected PBC should have an abdominal ultrasound as part of their baseline assessment, to exclude alternate aetiologies for cholestasis.	90%
2	All patients with suspected overlap features of autoimmune hepatitis (AIH) should have a liver biopsy with expert clinicopathological assessment to support diagnosis.	90%
3	All patients should receive UDCA as first-line treatment, at a dose of at least 13mg/kg/day.	90%
4	All patients with inadequate UDCA response or UDCA-intolerance should be considered for second-line treatment.	n/s
5	All patients should be evaluated for the presence of symptoms, in particular fatigue and pruritus, to ensure appropriate investigation and treatment.	80% (within the last 24 months)
6	All patients should have risk assessment for osteoporosis to optimise prevention of osteoporotic bone fractures. Treatment and follow-up should be according to national guidelines.	80% (within the last 5 years)
7	All patients with a bilirubin >50 µmol/L or evidence of decompensated liver disease should be discussed with a hepatologist in a transplant centre for timely consideration of liver transplantation.	90% (within 3 months)
8	All patients with cirrhosis should have surveillance for hepatocellular carcinoma (HCC).	n/s
9	All patients with clinically significant portal hypertension should have endoscopy screening for gastro-oesophageal varices.	n/s

Table S2: Comparison of National and International PBC guidelines by Major Societies

Audit Standard	BSG Guidelines 2018[1]	EASL Guidelines 2017[2]	AASLD Guidelines 2018[3]	APASL Guidelines 2021[4]
**Patients suspected to have PBC/AIH overlap syndrome should undergo liver biopsy.**	Overlap with AIH should be recognised as rare and, when suspected, liver biopsy with expert clinicopathological assessment is recommended to make the diagnosis.	PBC with features of AIH should be recognised as rare, and when suspected, liver biopsy with expert clinicopathological assessment, is recommended to make the diagnosis.	Liver biopsy to rule out concomitant AIH or other liver disease should be considered in PBC patients when the alanine aminotransferase activity is more than 5 times the upper limit of normal.	The diagnosis of PBC with AIH features could be made in PBC patients if two of the three following criteria are met: (1) moderate/severe interface hepatitis in liver histology (mandatory); (2) serum ALT/AST more than 5 times ULN; and (3) IgG level more than 1.3 times ULN or presence of ASMA.
**All patients should receive first-line therapy with UDCA at an adequate dose, or documented to be intolerant.**	Patients should be offered therapy with UDCA. UDCA at 13–15mg/kg/day is recommended for first-line use in all patients with PBC.	UDCA at 13–15 mg/kg/day is recommended for first-line use in all patients with PBC.	UDCA in a dose of 13 to 15 mg/kg/day orally is recommended for patients with PBC who have abnormal liver enzyme values regardless of histologic stage.	Oral UDCA (13 – 15mg/kg/day) should be standard therapy for all PBC patients.
**UDCA non-responders should be considered for second-line**	UDCA treated patients with an ALP >1.67x ULN and/or elevated bilirubin < 2 x ULN represent a group of high-risk patients	For patients with an inadequate response to UDCA, or for those intolerant to UDCA, consider the use of OCA.	Patients who are inadequate responders to UDCA should be considered for treatment with OCA, starting at 5 mg/day.	OCA should be added to UDCA therapy for PBC patients with an inadequate response to UDCA, or used in

**therapy with OCA or a fibrate.**	in whom there is randomised controlled trial evidence for the addition of second-line therapy.			monotherapy in those intolerant to UDCA.
**All patients should be assessed for pruritus and fatigue.**	Patients should be evaluated for the presence of symptoms, particularly fatigue and itch.	Patients should be evaluated for the presence of symptoms, particularly pruritus, sicca complex and fatigue.	The symptoms of PBC significantly impair quality of life and do not typically improve with UDCA or OCA treatment. Therefore, they warrant separate evaluation and treatment.	n/a
**All patients should have a risk assessment for osteoporotic fracture.**	Patients with PBC should have a risk assessment for osteoporosis. Treatment and follow-up should be according to national guidelines.	Patients should have a risk assessment for osteoporosis. Treatment and follow-up should be according to national guidelines.	Baseline and regular screening every 2 years using bone mineral density testing is appropriate.	Patients should be evaluated for osteoporosis, especially in postmenopausal women.
**All patients with a bilirubin > 50 should be discussed with a transplant centre.**	Patients with a bilirubin >50 µmol/L or evidence of decompensated liver disease should be discussed with a hepatologist linked to a transplant programme.	Patients with a bilirubin >50 µmol/L (3 mg/dl) or evidence of decompensated liver disease (variceal bleed, ascites, encephalopathy) should be discussed with a hepatologist linked to a transplant programme.	Patients with manifestations of end-stage PBC should be referred for liver transplantation when their Model for End-Stage Liver Disease score exceeds 14.	Liver transplant should be considered in patients with decompensated cirrhosis.

**Patients with cirrhosis should be under surveillance for HCC.**	In patients where cirrhosis is suspected, HCC surveillance should be carried out according to NICE guidelines.	Patients with suspected cirrhosis should have HCC surveillance according to EASL guidelines.	Regular screening for hepatocellular carcinoma with cross-sectional imaging at 6-month intervals is currently advised for patients with cirrhosis.	Close monitoring of HCC is recommended for patients with advanced-stage disease and non-responders to UDCA.
**Patients with clinically significant portal hypertension should be screen for gastro-oesophageal varices.**	Patients with suspected portal hypertension should be screened for gastro-oesophageal varices according to BSG guidelines.	Baveno-VI guidelines for screening and management of varices apply equally to patients with PBC.	Patients with suspected cirrhosis should undergo endoscopic screening for varices at the time of diagnosis.	Patients with features of portal hypertension should be screened for gastroesophageal varices.

1. British Society of Gastroenterology
2. European Association of Study of the Liver
3. American Association for the Study of Liver Diseases
4. Asian Pacific Association for the Study of the Liver

Table S3: Specific Questions with Indicative Timelines For Data Capture

What is the patient gender? (male/female)
What is the patient's current age?
Is the patient's weight recorded? (Y/N)
What is the patient weight in Kg?
In which clinic is the patient seen?<br>-Hepatology<br>-General Gastroenterology<br>-General Medicine<br>-Others (please specify)
When was the patient first diagnosed with PBC? (Y/N)
Did/does the patient have persistent elevation of serum ALP? (Y/N)
Does the patient have AMA detectable in serum? (Y/N)
Does the patient have PBC-specific ANA detectable in serum? (Y/N)
Has the patient had an USS of the liver at any point since the time of diagnosis? (Y/N)
Did the patient have an USS of the liver at diagnosis? (Y/N)
Has the patient ever had a liver biopsy? (Y/N)
Was the biopsy compatible with PBC? (Y/N)
Does the patient have PBC/AIH overlap syndrome? (Y/N)
Was the PBC/AIH overlap confirmed by a liver biopsy? (Y/N)
Is the patient currently treated with UDCA?<br>Is the current dose of UDCA recorded? (Y/N)<br>What is the total daily dose (mg/day)<br>Why is the patient not treated with UDCA? (If answered no to)<br>-Not offered by clinicians<br>-Declined by patient<br>-Intolerance of UDCA<br>-Unknown<br>-Other (Please specify?)
Was the patient referred to SLT MDT for alternative disease modifying treatment? (Y/N)
Why does the patient take <13mg/kg/day of UDCA?<br>-Optimal dose not offered by clinician<br>-Optimal dose declined by patient<br>-Intolerance of optimum dose<br>-Unknown<br>-Others
Has the patient taken UDCA for more than 12 months? (Y/N)
Based on the latest investigations, is the patient at high risk of disease progression? (Y/N)<br>*(based on the locally used definition of inadequate UDCA response, e.g. ALP > 1.67 x ULN after at least 12 months treatment)*
Was the patient referred to SLT MDT for consideration of second-line therapy? (Y/N)
Does the patient take any other disease-modifying treatment of PBC? (Y/N)
Which other disease-modifying treatment does the patient take?<br>-Obeticholic acid<br>-Bezafibrate<br>-Fenofibrate

-Budesonide<br>-Others (please specify)
Was this treatment recommended by the SLT MDT? (Y/N)
Do the clinic letters indicate that fatigue has been assessed within the last 24 months? (Y/N)<br>Do the clinic letters indicate that pruritus has been assessed within the last 24 months? (Y/N)<br>Did the patient have pruritus? (Y/N)<br>Does the patient currently receive treatment for PBC-related pruritus? (Y/N)<br>What is the treatment?<br>Is it clearly documented why the patient is not treated with Pruritus?
What treatment does the patient currently receive for PBC-related pruritus?<br>-Anti-histamines<br>-Cholestyramine<br>-Rifampicin<br>-Naltrexone<br>-Gabapentin<br>-Sertraline<br>-Others (what is the treatment – free text)
Is it clearly documented why the patient is not treated for pruritus? (Y/N)
Has the patient's risk of osteoporotic fracture been assessed within the last five years? (Y/N)<br>*(All types of risk assessment (FRAX score, DEXA scan, etc) as well as patient age and other health factors should be considered).*
Does the patient have a clinically significant risk of fracture? (Y/N)<br>(*As informed by the FRAX score or DEXA scan)*
Was appropriate action taken to reduce the risk of osteoporotic fracture? (Y/N)
Does the patient have cirrhosis? (Y/N)<br>*(Based on recent biopsy, imaging, elastography or supportive laboratory findings)*
Has the patient had an USS of the liver in the last 6 months? (Y/N)
Is it clearly documented why the patient did not have an USS? (Y/N)
Is the reason COVID-19 related delay? (Y/N)
Does the patient have clinically significant portal hypertension? (Y/N)<br>*(Based on Baveno criteria, the Newcastle Varices Score or locally agreed criteria)*<br>Has the patient had an OGD within the last 3 years? (Y/N)
Is it clearly documented why the patient has not had an OGD within the last 3 years? (Y/N)<br>Is the reason COVID-19 related delay? (Y/N)
IS the latest serum bilirubin >50umol/L? (Y/N)<br>Was the patient discussed with an LT centre? (Y/N)
Did this discussion take place within 3 months of the bilirubin first reaching 50umol/L? (Y/N)
Are there any other features of decompensated cirrhosis? (Y/N)<br>*(consider: UKELD score, evidence of ascites, hepatic encephalopathy or variceal bleed)*
Was the patient discussed with a LT Centre? (Y/N)

Blood tests ***(most recently available)*****:**
-Serum bilirubin
-Serum ALP
-Serum ALT
-Serum AST
-Serum Albumin
-Platelet count

Table S4: Overall Summary of Audit Performance

Performance standard	Number of patients meeting audit standard/total number patients (%)	Target (%)
**Diagnosis**		
Patients fulfilling diagnosis of PBC	8937/8968 (99.7)	n/a
Abdominal ultrasound scan at baseline*	2194/2491 (88.1)	90%
Liver biopsy undertaken	2856/8937 (32.0)	n/a
Biopsy compatible with PBC	2538/2856 (88.9)	n/a
Patients with a local diagnosis of PBC/AIH overlap	679/8937 (7.6)	n/a
PBC/AIH overlap diagnosis supported by liver biopsy	508/679 (74.8)	90%
**First-Line Therapy**		
Patients receiving UDCA as first-line therapy	7864/8937 (88.0)	n/a
Reason clearly documented for those not receiving UDCA	721/998 (72.2)	n/a
Patients not receiving UDCA due to intolerance	362/721 (50.2)	n/a
Patients receiving UDCA as first line therapy or documented intolerance	8226/8937 (92.0)	90%
Patients receiving UDCA dose of at least 13mg/kg	4203/6053 (69.4)	90%

**Second-Line Therapy**		
UDCA-untreated patients receiving second-line therapy	206/998 (20.6)	n/a
Patients with inadequate UDCA response according to local centre thresholds	2102/7395 (28.4)	n/a
Patients with inadequate UDCA response receiving second-line therapy	1074/2102 (51.1)	90%
**Symptom Assessment**		
Assessment of fatigue within the last 24 months	5052/8937 (56.5)	90%
Assessment of pruritus within the last 24 months	5522/8937 (61.8)	90%
**Osteoporosis Fracture Risk Assessment**		
Osteoporosis fracture risk assessment within the last 5 years	4883/8937 (54.6)	80%
Appropriate action taken in patients found to have a clinically significant risk of fracture	1447/1566 (92.4)	n/a
**Transplant Discussion**		
Patients with bilirubin > 50 µmol/L or hepatic decompensation discussed with a transplant centre	222/443 (50.1)	90%
Patients, aged below 70 years, with bilirubin > 50 µmol/L or evidence of hepatic decompensation discussed with a transplant centre	166/259 (64.1)	90%
**Surveillance**		
6 monthly HCC surveillance in patients with cirrhosis	1399/1947 (71.9)	90%
Surveillance of gastroesophageal varices in patients with clinically significant portal hypertension	695/905 (76.8)	90%

Table S5: Summary of Audit Performance According to Nation

Performance standard	Number of patients meeting audit standard/total number patients (%)			
	England	Wales	Scotland	NI
**Diagnosis**				
Abdominal ultrasound scan at baseline*	1929/2205 (87.5)	41/44 (93.2)	212/227 (93.4)	12/15 (80.0)
Liver biopsy undertaken	2509/7690 (32.6)	83/237 (35.0)	245/953 (25.7)	19/57 (33.3)
Biopsy compatible with PBC	2222/2509 (88.6)	75/83 (90.4)	223/245 (91.0)	18/19 (94.7)
Patients with local diagnosis of PBC/AIH overlap	582/7690 (7.6)	24/237 (10.1)	68/953 (7.1)	5/57 (8.8)
PBC/AIH overlap diagnosis confirmed by liver biopsy	433/582 (74.4)	21/24 (87.5)	49/68 (72.1)	5/5 (100)
**First-Line Therapy**				
Patients receiving UDCA as first line therapy	6742/7690 (87.7)	212/237 (89.5)	861/953 (90.3)	49/57 (86.0)
Reason clearly documented in patients not receiving UDCA	639/874 (73.1)	11/25 (44.0)	63/91 (69.2)	8/8 (100)
Patients documented to be UDCA-intolerant	320/639 (50.1)	5/11 (45.5)	30/63 (47.6)	7/8 (87.5)
Patients receiving UDCA as first line therapy or documented to be intolerant	7062/7960 (88.7)	217/237 (91.5)	891/953 (93.5)	56/57 (98.2)
Patients receiving UDCA of at least 13mg/kg	3533/5011 (70.5)	130/185 (70.3)	522/832 (62.7)	18/25 (72.0)
**Second-Line Therapy**				
UDCA-untreated patients receiving second-line therapy	117/874 (13.4)	3/25 (12.0)	20/91 (22.0)	6/8 (75.0)

Patients with inadequate UDCA response according to local centre thresholds	1826/6317 (28.9)	62/207 (30.0)	198/823 (24.1)	17/48 (35.4)
Patients with inadequate UDCA response receiving second-line therapy	927/1825 (50.8)	31/62 (50.0)	103/198 (52.0)	13/17 (76.5)
**Symptom Assessment**				
Assessment of fatigue within the last 24 months	4349/7690 (56.6)	134/237 (56.5)	527/953 (55.3)	42/57 (73.7)
Assessment of pruritus within the last 24 months	4758/7690 (61.9)	154/237 (65.0)	570/953 (59.8)	40/57 (70.2)
**Osteoporosis Fracture Risk Assessment**				
Risk assessment for osteoporosis within the last 5 years	4216/7690 (54.8)	106/237 (44.7)	531/953 (55.7)	30/57 (52.6)
Appropriate action taken in patients found to have a clinically significant risk of fracture	1249/1362 (91.7)	25/28 (89.3)	167/170 (98.2)	6/6 (100)
**Transplant Discussion**				
Patients with elevated bilirubin > 50 µmol/L or evidence of decompensation discussed with transplant centre	196/380 (51.6)	2/17 (11.8)	24/46 (52.2)	0/0 (0.0)
Patients, aged below 70, with elevated bilirubin > 50 µmol/L or evidence of decompensation discussed with transplant centre	143/216 (66.2)	2/11 (18.0)	21/32 (65.6)	0/0 (0.0)
**Surveillance**				
6 monthly US surveillance in patients with cirrhosis	1152/1639 (70.3)	53/72 (73.6)	184/224 (82.1)	10/12 (83.3)
Surveillance of gastroesophageal varices in patients with clinically significant portal hypertension	590/748 (78.9)	17/22 (77.3)	83/130 (63.8)	5/5 (100.0)

Table S6: Comparison of Audit Performance Between Specialist and Non-Specialist Centres

Performance standard	Number of patients meeting standard/total number patients (%)		OR	p-value†	95% CI Lower	95% CI Upper
	Specialist centres	Non-Specialist centres				
**Diagnosis**						
Abdominal ultrasound scan at baseline*	811/951 (85.3)	1118/1254 (89.2)	0.71	0.012	0.54	0.93
Liver biopsy undertaken	1410/3902 (36.1)	1099/3788 (29.0)	1.38	<0.001	1.26	1.53
Biopsy compatible with PBC	1276/1410 (90.5)	946/1099 (86.1)	1.54	<0.001	1.19	1.95
Patients with a local diagnosis of PBC/AIH overlap	277/3902 (7.1)	305/3787 (8.1)	0.87	0.121	0.73	1.04
PBC/AIH overlap diagnosis supported by liver biopsy	225/277 (81.2)	208/305 (68.2)	2.00	<0.001	1.35	3.03
**First-Line Therapy**						
Patients receiving UDCA as first line therapy	3466/3902 (88.8)	3276/3788 (86.5)	1.20	0.002	1.08	1.43
Reason clearly documented for those not receiving UDCA	327/414 (79.0)	312/460 (67.8)	1.78	<0.001	1.28	2.46
Patients not receiving UDCA due to intolerance	193/327 (59.0)	127/312 (40.7)	2.10	<0.001	1.51	2.91
Patients receiving UDCA as first line therapy or documented to be intolerant	3659/3902 (93.8)	3403/3788 (89.8)	1.70	<0.001	1.44	2.02
Patients receiving UDCA dose of at least 13mg/kg	1980/2664 (74.3)	1553/2347 (66.2)	1.48	<0.001	1.31	1.68
**Second-Line Therapy**						

UDCA-untreated patients receiving second-line therapy	106/414 (25.6)	71/460 (15.4)	1.88	<0.001	1.33	2.68
Patients with inadequate UDCA response according to local centre thresholds	1053/3298 (31.9)	772/3019 (25.6)	1.36	<0.001	1.22	1.52
Patients with inadequate UDCA response receiving second-line therapy	699/1053 (66.4)	228/772 (29.6)	4.69	<0.001	3.82	5.76
**Symptom Assessment**						
Assessment of fatigue within the last 24 months	2212/3902 (56.7)	2137/3788 (56.4)	1.01	0.82	0.92	1.11
Assessment of pruritus within the last 24 months	2547/3902 (65.3)	2211/3788 (58.4)	1.34	<0.001	1.22	1.47
**Osteoporosis Fracture Risk Assessment**						
Osteoporosis fracture risk assessment within the last 5 years	2343/3902 (60.0)	1873/3788 (49.4)	1.53	<0.001	1.40	1.68
Appropriate action taken in patients found to have a clinically significant risk of fracture	641/698 (91.8)	608/664 (91.6)	1.04	0.92	0.69	1.55
**Transplant Discussion**						
Patients with bilirubin > 50 µmol/L or hepatic decompensation discussed with a transplant centre	118/188 (62.8)	78/192 (40.6)	2.46	<0.001	1.60	3.80
Patients, aged below 70 years, with bilirubin > 50 µmol/L or hepatic decompensation discussed with a transplant centre	83/109 (76.1)	60/107 (56.1)	2.49	0.002	1.34	4.69
**Surveillance**						
6 monthly HCC surveillance in patients with cirrhosis	596/825 (72.2)	556/814 (68.3)	1.80	<0.001	1.26	2.58
	284/366 (77.6)	306/382 (80.1)	0.86	0.42	0.60	1.24

Surveillance of gastroesophageal varices in patients with clinically significant portal hypertension						

†Fisher's exact test
*Due to varying access of historical radiology, this analysis was only performed on patients diagnosed on/after 1st January 2017

Table S7: Summary of Audit Performance Across Regions in England

Performance standard	Number of patients meeting audit standard/total number patients (%)								
	North East	North West	Yorkshire	West Midlands	East Midlands	East of England	London	South East	South West
**Diagnosis**									
Abdominal ultrasound scan at baseline*	120/127 (94.5)	191/217 (88.0)	196/213 (92.0)	196/220 (89.0)	146/178 (82.0)	311/354 (87.9)	283/311 (91.0)	321/387 (82.9)	165/198 (83.3)
Liver biopsy undertaken	172/513 (33.5)	233/792 (29.4)	264/814 (32.4)	261/886 (29.5)	194/602 (32.2)	292/1022 (28.6)	470/1096 (42.9)	413/1153 (35.8)	210/812 (25.9)
Biopsy compatible with PBC	161/172 (93.6)	197/233 (84.6)	242/264 (91.7)	215/261 (82.4)	167/194 (86.1)	265/292 (90.8)	406/470 (86.4)	413/413 (100.0)	190/210 (90.5)
Patients with a local diagnosis of PBC/AIH overlap	37/513 (7.2)	54/792 (6.8)	76/814 (9.3)	51/886 (5.8)	44/602 (7.3)	55/1022 (5.4)	87/1096 (7.9)	111/1153 (9.6)	67/812 (8.2)
PBC/AIH overlap diagnosis supported by liver biopsy	28/37 (75.7)	38/54 (70.3)	65/76 (85.5)	27/51 (52.9)	34/44 (77.3)	38/55 (69.1)	63/87 (72.4)	85/111 (76.6)	55/67 (82.1)

**First-Line Therapy**									
Patients receiving UDCA as first-line therapy	465/513 (90.6)	696/792 (87.8)	742/814 (91.2)	760/886 (85.7)	512/602 (85.0)	911/1022 (89.1)	970/1096 (88.5)	1004/1153 (87.0)	682/812 (84.0)
Reason clearly documented in patients not receiving UDCA	40/47 (85.1)	68/84 (80.9)	57/68 (83.8)	67/112 (59.8)	68/88 (77.3)	83/109 (76.1)	98/121 (81.0)	74/134 (55.2)	84/111 (75.6)
Patients documented as being UDCA-intolerant	23/40 (57.5)	38/68 (55.9)	35/57 (61.4)	39/67 (58.2)	26/68 (38.2)	29/83 (34.9)	56/98 (57.1)	38/74 (51.3)	36/84 (42.9)
Patient receiving UDCA as first-line therapy or documented to be intolerant	488/513 (95.1)	734/792 (92.6)	777/814 (95.4)	799/886 (90.2)	538/602 (89.4)	940/1022 (91.9)	1026/1096 (93.6)	1042/1153 (90.4)	718/812 (88.4)
Taking UDCA at dose of at least 13mg/kg	282/375 (75.2)	313/464 (67.4)	391/574 (68.1)	443/633 (70.0)	148/256 (57.8)	536/735 (72.9)	526/738 (71.3)	623/843 (73.9)	271/393 (69.0)
**Second-Line Therapy**									
UDCA-untreated patients receiving second-line therapy	12/47 (25.5)	24/84 (28.6)	10/68 (14.7)	24/112 (21.4)	13/88 (14.8)	17/109 (15.6)	43/121 (35.5)	18/111 (16.2)	16/134 (11.9)
Patients with inadequate UDCA response according to local centre thresholds	136/433 (31.4)	203/636 (31.9)	166/697 (23.8)	230/710 (32.4)	172/492 (34.9)	186/855 (21.7)	284/924 (30.7)	275/919 (29.9)	173/651 (26.6)

Patients with inadequate UDCA response receiving second-line therapy	84/136 (61.8)	84/203 (41.4)	65/166 (39.1)	137/230 (59.6)	93/172 (54.0)	114/186 (61.2)	153/284 (53.9)	121/275 (44.0)	76/173 (43.9)
**Symptom Assessment**									
Assessment of fatigue within the last 24 months	360/513 (70.1)	389/792 (49.1)	389/814 (47.8)	512/886 (57.8)	342/602 (56.8)	590/1022 (57.7)	657/1096 (59.9)	660/1153 (57.2)	450/812 (55.4)
Assessment of pruritus within the last 24 months	396/513 (77.2)	419/792 (52.9)	482/814 (59.2)	523/886 (59.0)	342/602 (56.8)	618/1022 (60.5)	779/1096 (71.1)	726/1153 (63.0)	473/812 (58.3)
**Osteoporosis Fracture Risk Assessment**									
Osteoporosis fracture risk assessment within the last 5 years	350/513 (68.2)	404/792 (51.0)	520/814 (63.9)	298/886 (33.6)	269/602 (44.7)	632/1022 (61.8)	628/1096 (57.3)	693/1153 (60.1)	422/812 (52.0)
Appropriate action taken in patients found to have a clinically significant risk of fracture	92/106 (86.8)	119/134 (88.8)	142/154 (92.2)	65/71 (91.5)	72/75 (96.0)	148/169 (87.6)	222/240 (92.5)	238/251 (94.8)	151/162 (93.2)
**Transplant Discussion**									
Patients with bilirubin > 50 µmol/L or hepatic decompensation discussed with a transplant centre	7/12 (50.0)	8/34 (23.5)	19/26 (73.0)	63/81 (77.7)	9/31 (29.0)	32/54 (59.2)	33/49 (61.2)	15/63 (23.8)	10/30 (33.3)

Patients, aged below 70 years, with bilirubin > 50 µmol/L or hepatic decompensation discussed with a transplant centre	4/4 (100)	7/23 (30.4)	17/20 (85.0)	51/56 (91.1)	6/13 (46.1)	24/31 (77.4)	17/26 (65.4)	10/28 (35.7)	9/16 (56.3)
**Surveillance**									
6 monthly HCC surveillance in patients with cirrhosis	74/90 (82.2)	113/169 (66.8)	106/147 (72.1)	176/249 (70.7)	71/110 (64.5)	166/243 (68.3)	194/272 (71.3)	141/215 (65.6)	111/144 (77.1)
Surveillance of gastroesophageal varices in patients with clinically significant portal hypertension	23/33 (69.7)	47/62 (75.8)	82/101 (81.2)	98/122 (80.3)	29/46 (63.0)	83/97 85.6)	75/100 (75.0)	83/107 (77.6)	70/80 (87.5)

Fig. S1: Summary of Audit Performance Across Regions in England

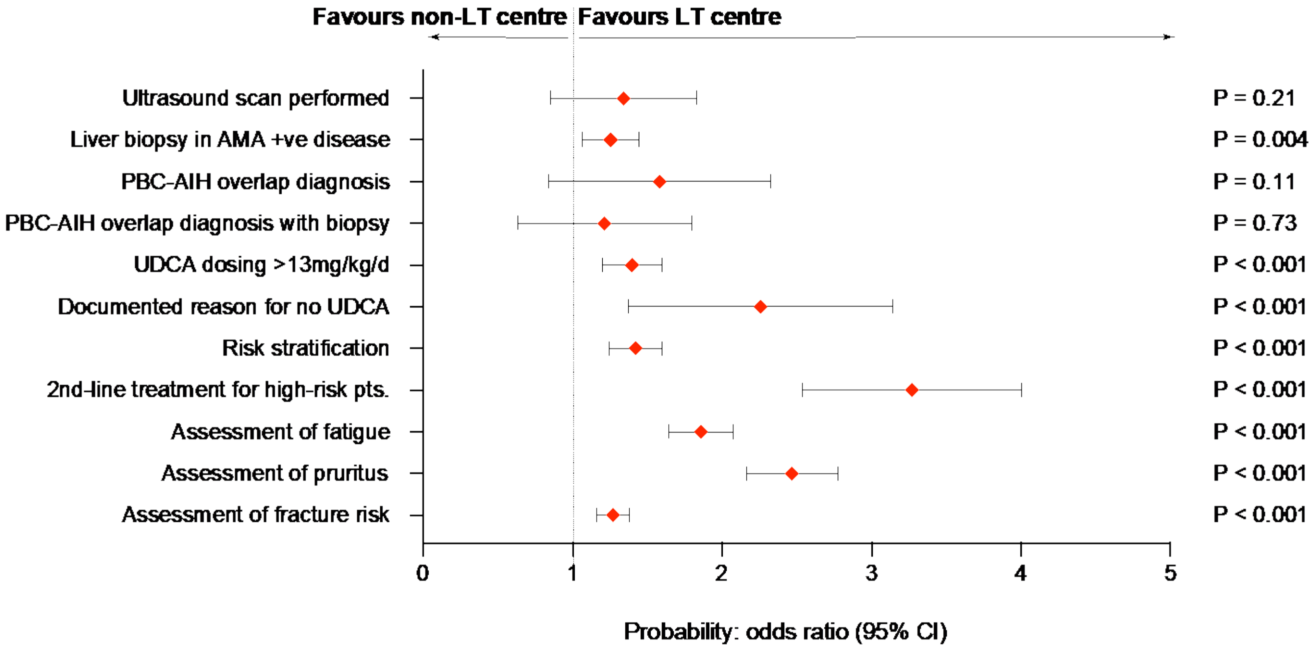

Forrest plot indicating the probability of a centre meeting a particular audit standard according to status as a liver transplant versus non-transplant unit.
